# Neuronal activity and amyloid-β promote tau seeding in the entorhinal cortex in Alzheimer’s disease

**DOI:** 10.1093/brain/awaf374

**Published:** 2025-10-07

**Authors:** Christoffer G Alexandersen, Dani S Bassett, Alain Goriely, Pavanjit Chaggar

**Affiliations:** Department of Bioengineering, School of Engineering and Applied Science, University of Pennsylvania, Philadelphia, PA 19104, USA; Mathematical Institute, University of Oxford, Oxford OX4 6GG, UK; Department of Bioengineering, School of Engineering and Applied Science, University of Pennsylvania, Philadelphia, PA 19104, USA; Department of Electrical & Systems Engineering, School of Engineering and Applied Science, University of Pennsylvania, Philadelphia, PA 19104, USA; Department of Physics & Astronomy, School of Arts & Sciences, University of Pennsylvania, Philadelphia, PA 19104, USA; Departments of Neurology & Psychiatry, Perelman School of Medicine, University of Pennsylvania, Philadelphia, PA 19104, USA; The Neuro, Montreal Neurological Institute, McGill University, Montreal, QC H3A 0G4, Canada; Santa Fe Institute, Santa Fe, NM 87501, USA; Mathematical Institute, University of Oxford, Oxford OX4 6GG, UK; Mathematical Institute, University of Oxford, Oxford OX4 6GG, UK; Clinical Memory Research, Lund University, Lund 221 00, Sweden

**Keywords:** prion-like, synaptic activity, neurodegenerative disease, protein aggregation, disease progression, neuroimaging

## Abstract

The entorhinal cortex is the first region to develop tau pathology in Alzheimer’s disease and primary age-related tauopathy, yet the reasons for this selective vulnerability remain unclear. We developed a computational model in which neuronal activity and amyloid-β (Aβ) modulate tau transport, hypothesizing that this mechanism explains entorhinal vulnerability to early tau pathology.

The model combines structural connectivity with either neuronal activity (measured by FDG PET) or Aβ burden (measured by Aβ PET). We analysed Alzheimer’s Disease Neuroimaging Initiative (ADNI) data comprising 527 FDG PET scans (mean age 71.8 years; 174 cognitively normal, 293 mild cognitive impairment, 60 Alzheimer’s disease) and 1244 Aβ PET scans (mean age 72.4 years; 501 cognitively normal, 588 mild cognitive impairment, 155 Alzheimer’s disease). From these, 253 FDG–tau and 453 Aβ–tau PET pairs were used in regression analyses. Key results were replicated in the Harvard Aging Brain Study (HABS; 300 FDG, 348 Aβ, 116 FDG–tau and 255 Aβ–tau pairs).

Both FDG- and Aβ-based models consistently identified the entorhinal cortex as a primary tau seeding region in ADNI (FDG: *z* ≈ 4.6–4.9, *P* < 0.0066; Aβ: *z* ≈ 4.0–8.7, *P* ≤ 0.011) and in HABS (FDG: *z* = 5.7, *P* = 0.030; Aβ: *z* = 6.0, *P* = 0.0018). Simple linear regression showed modest associations between model-derived seeding and empirical entorhinal tau in ADNI (FDG: *β* = 6.7, *P* = 0.0039; Aβ: *β* = 11.3, *P* < 0.001), which remained significant after adjustment for age, sex, and APOE4 status (FDG: *β* = 7.1, *P* < 0.001; Aβ: *β* = 9.7, *P* < 0.001). Aβ-based associations replicated in HABS (*β* = 3.3, *P* < 0.001), while FDG-based correlations were not detectable in this predominantly cognitively normal cohort (*β* = −0.43, *P* = 0.80; power = 49%).

These findings support a mechanistic role for neuronal activity and Aβ in initiating tau pathology, with the entorhinal cortex consistently emerging as highly vulnerable. Our computational model reliably identifies this region as the epicentre of pathology, supporting the idea that brain-wide patterns of neuronal activity and amyloid burden determine where tau pathology begins.

## Teaser

The entorhinal cortex is the first region to develop tau pathology in Alzheimer’s disease, but the basis for its vulnerability remains unclear. Using neuroimaging data and a computational model, Alexandersen *et al.* show that brain-wide patterns of neuronal activity and amyloid burden drive early tau accumulation in this region.

## Introduction

Tau pathology is a defining characteristic of Alzheimer’s disease (AD) and primary age-related tauopathy, with the medial temporal lobe typically being the first region to exhibit tau accumulation, particularly in the entorhinal cortex.^1-5^ Despite consistent evidence demonstrating tau pathology in the entorhinal cortex during the initial stages of AD, we currently have a poor understanding of why the entorhinal cortex is the initial site of tau pathology. Gaining such an understanding may be crucial for the development of early interventions to prevent or delay tau-related pathology in AD.

Toxic tau is believed to propagate across the brain primarily via cell-to-cell transmission; however, the mechanisms through which this transport process is regulated are unclear. Experiments in transgenic animal models have demonstrated that misfolded tau proteins, either injected into the brain or overexpressed in the entorhinal cortex, result in local tau accumulation and propagation to nearby regions.^6-10^ This evidence is complemented by studies using intracranial injections of synthetic tau fibrils or human brain-derived pathological tau into mouse models to show that tau aggregates can propagate through axonal connections.^11-14^ This process has been further validated using human neuroimaging studies, in which models describing trans-synaptic spread of tau pathology accurately predict tau progression.^15-17^ However, other factors such as neuronal activity and amyloid-β (Aβ) also modulate the tau transport process.

*In vitro* studies have shown that greater neuronal activity results in increased interneuronal tau transport,^18-20^ and animal models combining Aβ and tau pathology show enhanced propagation when Aβ pathology is present.^21,22^ These mechanisms may indeed be linked, since Aβ has been shown to induce neuronal hyperexcitability in rat slices and *in vivo* mouse models,^23-25^ suggesting that Aβ may exert its influence on tau spreading through neuronal hyperexcitability.

Further confounding the relationship between Aβ and tau is their spatial properties—while tau pathology in primary age-related tauopathy and AD begins in the entorhinal cortex,^1-3,5,26^ early Aβ depositions are mainly present in the neocortex,^26,27^ suggesting that the effects of Aβ on tau may be non-local. Overall, the relationship between Aβ, tau, and neuronal activity is complex,^28^ and it remains unclear whether Aβ deposition directly enhances tau seeding or if it modulates tau spread through other means, such as Aβ-induced hyperexcitability. Particularly puzzling is the fact that tau accumulation may occur in the medial temporal lobe without the involvement of Aβ deposition, such as in primary age-related tauopathy.^1-3,5^ Thus, Aβ deposition is not necessary for early tau accumulation in the medial temporal lobe but is necessary for tau to spread from the medial temporal lobe to neocortical regions.

In a recent analysis of a model coupling neuronal activity to interneuronal protein transport,^29^ we showed that activity-dependent transport of physiological tau could elevate local concentrations beyond a critical threshold, thereby initiating pathological aggregation, and thus determine where pathology begins. This process provides a mechanistic explanation for how regional patterns of neuronal activity might determine where tau pathology first emerges. In the present study, we simplify this prior model and integrate it with empirical PET data from the Alzheimer’s Disease Neuroimaging Initiative (ADNI) to test whether activity- or Aβ-modulated transport of tau can explain regional vulnerability to early tau pathology. Specifically, we investigate whether the spatial distribution of (i) neuronal activity [from fluorodeoxyglucose (FDG) PET]; or (ii) amyloid burden (from Aβ PET) predicts initial tau seeding in the entorhinal cortex, consistent with the known sequence of primary age-related tauopathy and AD pathology.

Using FDG PET imaging to inform our computational model, we demonstrate that brain-wide activity patterns predict tau seeding in the medial temporal lobe. The predicted seeding of tau in the medial temporal lobe is consistent across subject classifications based on diagnosis and amyloid status. Furthermore, under the assumption that Aβ increases tau transport, we show that Aβ deposition patterns predict the entorhinal cortex to be the most vulnerable region to tau seeding, with a particularly strong bias toward entorhinal seeding. We also show that the model predictions of tau seeding in early Braak stage regions are statistically significant upon re-shuffling PET signals by region for the FDG- and Aβ-based models. Moreover, subject-level analysis confirms that predicted seeding concentrations correlate significantly with empirical tau deposition, supporting the hypothesis that neuronal activity and amyloid deposition shape early tau pathology. These findings were replicated in an independent cohort from the Harvard Aging Brain Study (HABS): the entorhinal cortex again emerged as a primary seeding site with high significance for both FDG- and Aβ-based models. Subject-level correlations also generalized for Aβ-based predictions, but not for FDG, possibly due to cohort differences and limited statistical power.

Collectively, these results support the hypothesis that brain-wide gradients of neuronal activity contribute to tau accumulation in the medial temporal lobe and that Aβ deposition further amplifies seeding in the entorhinal cortex. Together, they offer a causal account for why tau pathology begins in this region in both primary age-related tauopathy and AD.

## Materials and methods

### PET data processing

We used PET imaging and diagnostic data from ADNI (adni.loni.usc.edu), including FDG, Aβ (florbetapir), and tau (flortaucipir) PET scans.^30^ No partial volume correction was applied. For Aβ and tau, we used the fully processed ADNI summary standardized uptake value ratio (SUVR) tables based on the Desikan-Killiany atlas.^31^ Each subject contributed a single Aβ and tau PET scan. Participants were stratified by clinical diagnosis using the earliest available Aβ scan: cognitively normal (CN), mild cognitive impairment (MCI) or AD. Additional stratification was done by biomarker status (Aβ/tau status) using the earliest available Aβ and tau scan pair within ±12 months. Aβ SUVRs were calculated using the whole cerebellum as the reference region, and tau SUVRs were calculated using the inferior cerebellum. Aβ positivity was defined as a cortical SUVR > 1.11 (20 Centiloids; ADNI cortical summary region: bilateral frontal, anterior/posterior cingulate, lateral parietal, lateral temporal regions)^32^ and tau positivity was defined based on SUVR thresholds for two regions: a medial temporal lobe composite SUVR > 1.375 (entorhinal cortex and amygdala) and a neocortical composite SUVR > 1.395 (inferior and middle lateral temporal cortex), using an inferior cerebellum reference. These thresholds were derived using Gaussian mixture modelling.^33^ We analysed the following biomarker groups: Aβ^−^ (amyloid-negative), Aβ^+^τMTL^−^τNEO^−^ (amyloid-positive, tau-negative), Aβ^+^τMTL^+^τNEO^−^ (amyloid-positive, tau-positive in the medial temporal lobe, tau-negative in the neocortex) and Aβ^+^τMTL^+^τNEO^+^ (amyloid-positive, tau-positive in the medial temporal lobe and neocortex). The group Aβ^+^τMTL^−^τNEO^+^ was excluded due to low sample size. Demographic details are summarized in Table 1.

**Table 1:** Demographic and clinical characteristics across all cohorts

	ADNI biomarker				ADNI diagnosis			ADNI regression	HABS overall	HABS regression
	Aβ^−^	Aβ^+^τMTL^-^τNEO^−^	Aβ^+^τMTL^−^τNEO^−^	Aβ^+^τMTL^−^τNEO^−^	CN	MCI	AD	All	All	All
**FDG**										
*n*	285	47	37	88	174	293	60	253	300	116
Age	70.4	74.0	73.9	72.0	72.0	71.2	73.8	74.3	73.1	72.0
Gender	44%	40%	43%	50%	55%	40%	40%	40%	60%	57%
Education	16.5	15.9	16.8	15.6	16.5	16.3	15.6	16.1	15.8	15.9
APOE4	22%	44%	65%	80%	27%	36%	57%	46%	28%	30%
CN	33%	11%	3%	1%	–	–	–	9%	76%	74%
MCI	63%	83%	81%	64%	–	–	–	73%	13%	14%
AD	4%	6%	16%	35%	–	–	–	18%	0%	0%
Cent.	−0.1	52.7	70.4	99.5	17.5	36.4	81.8	46.5	–	–
Cent. SD	12.4	35.0	37.3	31.7	34.6	46.4	50.8	51.9	–	–
**Aβ**										
*n*	686	131	43	84	501	588	155	453	348	255
Age	71.4	71.9	73.2	72.3	72.4	71.9	74.4	75.2	71.6	76.6
Gender	47%	52%	53%	54%	56%	44%	44%	50%	60%	61%
Education	16.5	16.6	16.3	15.8	16.5	16.1	15.5	16.4	15.9	16.2
APOE4	21%	44%	68%	62%	29%	45%	63%	36%	28%	27%
CN	34%	41%	23%	11%	–	–	–	57%	78%	93%
MCI	63%	57%	70%	74%	–	–	–	31%	12%	5%
AD	3%	2%	7%	15%	–	–	–	11%	0%	0%
Cent.	−1.5	36.9	62.1	89.2	19.9	414	80.8	26.9	–	–
Cent. SD	120.6	33.3	41.2	34.7	36.4	49.2	46.1	43.2	–	–

Values are shown separately for FDG (*top*) and Aβ (*bottom*) PET cohorts. Reported measures include mean years of age, years of education, gender distribution (proportion female), APOE4-carrier proportion, diagnostic group proportions (CN, MCI, AD) and Centiloid means and standard deviations. Aβ = amyloid-β; AD = Alzheimer’s disease; ADNI = Alzheimer’s Disease Neuroimaging Initiative; Cent. = Centiloids; CN = cognitively normal; FDG = fluorodeoxyglucose; HABS = Harvard Aging Brain Study; MCI = mild cognitive impairment; SD = standard deviation; τMTL\NEO = tau in the medial temporal lobe\neocortex.

FDG PET scans were obtained in preprocessed form (frame-averaged and smoothed) and paired with raw structural MRI scans for spatial normalization. Skull-stripping and registration to Montreal Neurological Institute (MNI) space were performed using Advanced Normalization Tools (ANTs; SyN algorithm). FDG images were normalized by the mean pons signal to compute SUVRs in cortical regions of interest (ROIs) defined by the Desikan-Killiany atlas, parcellated in MNI ICBM152 template space via FreeSurfer.^34^ Subjects contributed a single FDG PET scan and were stratified both by diagnosis at the time of the scan and by biomarker status, using FDG scans matched within ±12 months of Aβ/tau PET pairs. We analysed the same biomarker groupings as for Aβ PET. As with Aβ data, the small Aβ^+^τMTL^−^τNEO^+^ group was excluded.

To correct for signal instability in regions prone to segmentation artefacts (frontal pole, temporal pole and banks of the superior temporal sulcus), we replaced their SUVRs with the average of themselves and neighbouring regions, following previous work.^35^ This correction was applied consistently across FDG and Aβ PET (Supplementary Table 1).

Further details on data processing, including for the replication cohort from the Harvard Aging Brain Study (HABS), are provided in the Supplementary material.^36^

### Structural connectivity

The weighted adjacency matrix used for the computational modelling was obtained from the diffusion-weighted MRI images of 150 random young healthy participants in the Human Connectome Project.^37,38^ We used the probtrackx algorithm from FSL,^39^ with the default parameters and 1000 samples per voxel, sampled randomly from a sphere around the voxel centre. The average adjacency matrix from these individuals was calculated, which was then symmetrized and divided by the maximum value. We applyied a cut-off filter to the normalized averaged adjacency matrix of > 0.01 to mitigate against spurious connections.

### Braak staging

In our analyses, Braak stages 1–6 were defined according to the regional groupings shown in Table 2. However, we note that the hippocampus has been shown to have poor signal quality due to off-target binding in the adjacent choroid plexus,^40^ and therefore we excluded the hippocampus when comparing model-derived seeding predictions with empirical tau SUVR in Braak stages 2/3.

**Table 2:** Regions (ipsi- and contralateral) included in each Braak stage

Braak stage	Regions included
1	Entorhinal cortex
2/3	Lingual gyrus, fusiform gyrus, parahippocampal gyrus, hippocampus, amygdala
4	Rostral/anterior cingulate, caudal/anterior cingulate, posterior cingulate, isthmus cingulate, temporal pole, inferior temporal gyrus, middle temporal gyrus, insula
5	Lateral orbitofrontal cortex, pars orbitalis, frontal pole, medial orbitofrontal cortex, pars triangularis, pars opercularis, rostral middle frontal gyrus, superior frontal gyrus, caudal middle frontal gyrus, supramarginal gyrus, superior parietal lobule, inferior parietal lobule, precuneus, lateral occipital cortex, banks of the superior temporal sulcus, superior temporal gyrus, transverse temporal gyrus
6	Precentral gyrus, paracentral lobule, postcentral gyrus, cuneus, pericalcarine cortex

### Computational modelling

Mathematical models have been used to study the prion-like spread of proteins in various neurodegenerative diseases.^41-45^ These models simulate the spread and increase of protein pathology through structural connectomes—brain networks that map the physical architecture of the brain, inferred from diffusion tensor or weighted imaging and tractography. These computational models show promising agreement with empirical data.^17,33^ The simplest class of spreading models are linear diffusion models, which describe a transport process across a network.^41^ These linear diffusion models may be complemented with non-linear dynamics, often representing local prion-like replication within each brain region.^43^

In this study, however, we are not modelling the progression of tau pathology throughout the entire disease but rather the diffusion of early tau seeds before disease initiation. Our goal was to predict which regions are most susceptible to early tau seeding. Since protein densities are low in the early stage, non-linear effects do not yet play a role and linear effects dominate. Hence, we used a linear diffusion model describing the mass-conserving transport of protein seeds between regions and local production and decay of tau seeds. Moreover, we weighted the axonal transport of the protein by a specific transport process, which, in our study, will either be (i) neuronal activity; or (ii) Aβ deposition. Since this effect is a modification of a passive diffusion process, we refer to it as an anomalous transport process (generated by neuronal activity or amyloid deposition).

The connectome is represented by the *N* × *N* weighted adjacency matrix ***W***, where each node corresponds to a brain region, and edges are weighted as *W_ij_* = *n_ij_*, the number of fibres from region *j* to region *i*. The regular diffusive transport between different nodes (brain regions) is usually described using the graph Laplacian ***L*** with entries


(1) $$L_{ij}=-W_{ij}+\delta_{ij}\sum_{k=1}^{N}W_{kj},\;\;i,\;j=1,\;\ldots ,\;N,$$

where *δ_ij_* is the Kronecker symbol. In our case, however, we scaled the axonal transport by an anomalous transport process. Each node has an ‘anomaly level’ associated with it *A_i_*, and we collected these in a diagonal *N* × *N* matrix ***A*** = diag(*A*_1_*, …, A_n_*). We then defined an anomaly-scaled adjacency matrix W = ***W***(***I*** +*ɛA*), where ***I*** is the *N* × *N* identity matrix and *ɛ* the strength of the anomaly. The Laplacian L built from *W* is then given by


(2) L=L(I+εA)

Here, we will consider a protein species ***u*** ∈R*^N^*, namely tau seeds, that is being naturally produced and degraded in each node (brain region) and transported across edges (axonal connections), where the transport is accelerated by the anomalous transport process. This dynamical process is modelled by


(3) $${\dot{u}}_{i}=-\rho \sum_{j=1}^{N}L_{\mathrm{ij}}(1+\varepsilon A_{j})u_{j}+k-\lambda u_{i},\;\;i=1,\;\ldots ,\;N,$$

where *ρ >* 0 is the transport coefficient, *k >* 0 is the protein production rate, and *λ >* 0 is the protein degradation rate. We assumed that the production and degradation rates were the same across all regions as we focused on the transport process.

Note that as *ɛ* → 0, we recover the traditional transport process that depends only on the structural connectivity. As *ɛ* increases, so does the anomalous transport process’s impact on the protein spreading. We gained more insight into the impact of the anomalous transport process by considering the eigenvector corresponding to the zero eigenvalue of the anomaly-weighted Laplacian L. A graph Laplacian with a single, fully connected component always has a zero eigenvalue with a 1-dimensional eigenspace. The 0-eigenvector is, in these cases, the stationary solution (up to scaling) of d***x***/d*t* = −***Lx***. If ***v*** is the 0-eigenvector of the Laplacian built from the structural connectivity ***L*** alone, then the 0-eigenvector of the anomaly weighted Laplacian L has components *v_i_*/(1 *+ ɛA_i_*). In other words, the anomalous transport process ***A*** leads to higher accumulation in regions with low *A_i_* and lower accumulation in regions with high *A_i_*. Hence, a region with low activity surrounded by neighbours with high activity will naturally accumulate proteins.

Here, we are interested in the impact of neuronal activity and amyloid deposition on tau seeding. Therefore, we focused on parameter regimes in which the production of tau seeds is small compared to protein transport. In such a parameter regime, the tau seeding predictions will be mainly impacted by the anomalous transport process, which will either be neuronal activity or amyloid deposition. We started all regions with the same level of protein concentration ***u***(0) = 0.1, so that there was no built-in bias towards any particular region, apart from how the PET data informs the computational model. The parameters were chosen manually to ensure that transport dominates over local production: *ɛ* = 5*, ρ* = 1.0, *k* = 0.02 and *λ* = 0.2, which also ensures a production-to-degradation ratio consistent with the initial conditions. For the time series simulations, we re-scaled time by a factor of 0.7 to display the trajectory over a 15-unit time span. We investigated the impact of varying *ɛ* in the Supplementary material. All simulations and analyses were written in the Julia programming language, and the differential equations were solved using the Tsitouras 5th-order method from the DifferentialEquations Julia package with an absolute and relative tolerance of 10^−10^. Although our model was designed to test early tau seeding mechanisms, we included PET scans from individuals at varying stages of disease, including those with tau pathology beyond the medial temporal lobe. This was done deliberately to evaluate whether the spatial gradients of FDG and Aβ deposition—and thus the model’s predicted seeding patterns—change across disease stages.

### Statistical analysis

#### Assessing group differences in predicted tau seeding

To determine whether tau seeding predictions differ between subjects based on diagnosis and biomarker (Aβ and tau) status, we compared the average predicted seeding concentrations between groups. We used each individual’s FDG and Aβ PET SUVRs to predict their personalized seeding concentration in the entorhinal cortex, generating a distribution for each group. We then used two-sided Welch’s *t*-tests to assess whether the mean predicted seeding concentrations differ significantly between the groups. The significance levels are defined as *P* < 0.05, *P* < 0.01 and *P* < 0.001. A Bonferroni correction was applied to adjust the significance levels for multiple comparisons to the distributions.

#### Assessing the significance of predicted tau seeding regions

Using our computational model, we predicted seeding regions and tested their significance against a null model generated by randomizing regional PET SUVRs. The null hypothesis is that patterns of neuronal activity and amyloid deposition do not contribute to tau seeding in early Braak regions, regardless of diagnosis or biomarker status. The alternative hypothesis is that regional neuronal activity or amyloid deposition does contribute to tau seeding in early Braak regions. To evaluate this, we shuffled PET SUVRs across regions and generated seeding predictions from the randomized inputs, repeating this process 10 000 times. The resulting tau concentrations across all regions and trials formed a distribution of null values. We defined a concentration threshold beyond which a region is considered seeded by the model. This seeding threshold was set as the midpoint of the largest gap between any two adjacent null concentrations, after restricting to the upper half of the ranked null distribution (i.e. values above the median; Supplementary Figs 4 and 10). This defines a threshold that separates higher null concentrations based on the largest observed jump in the upper half of the distribution.

For statistical validation, we performed two tests. First, we counted the number of seeding regions belonging to Braak stage 1 (bilateral entorhinal cortex) in our non-shuffled modelling predictions. Using the null model, we calculated the probability of observing these many or more seeded regions in Braak stage 1 with randomly shuffled regional PET values. This probability served as the *P*-value for Braak stage 1 predictions. Second, the same procedure was repeated but seeded regions belonging to Braak stages 1–3 were counted. The significance levels are defined as *P* < 0.05, *P* < 0.01 and *P* < 0.001.

These *P*-values allowed us to assess the probability that the regional patterns of neuronal activity and amyloid deposition observed in the study subjects contribute to tau seeding in early Braak stages under the null hypothesis that their patterns of regional values are unrelated to tau seeding.

#### Assessing subject-level correlations between model-predicted seeding and early tau deposition

To examine the relationship between neuronal activity, amyloid deposition, and early tau seeding across subjects, we performed simple, least-squares linear regression. The explanatory variables were the asymptotic model-derived seeding concentrations based on subject-level FDG and Aβ PET patterns, and the predicted variables were subject-level empirical tau PET SUVRs, where both variables were averaged over the bilateral entorhinal cortex (or, alternatively Braak stage 2/3 regions as shown in the Supplementary material). For each scan, we required that the FDG or Aβ PET scans were taken within ±12 months of the tau PET scan. For the ADNI cohort, we picked the earliest eligible FDG-tau (*n* = 253) and Aβ-tau (*n* = 453) scan pair available per participant. For the HABS cohort, we instead picked the latest FDG-tau (*n* = 116) and Aβ-tau (*n* = 255) scan pair available, as all participants entered this cohort as asymptomatic. Only one FDG-tau and Aβ-tau scan pair were included per participant. The demographics of the cohort subsets used in the regression analyses are presented in Table 1. We tested the null hypothesis that the regression slope was equal to zero (two-sided Student’s *t*-test), with significance levels defined as *P* < 0.05. Pearson correlation coefficients (*r*-values) were computed to quantify the strength of the association between the predicted and observed tau seeding across subjects. Because flortaucipir shows off-target binding in the hippocampus, we excluded hippocampal ROIs when comparing model output to PET data for Braak 2/3.

To account for subject-level variability, we performed multiple linear regression adjusting for age, sex, and APOE4 carrier status. These covariates were included as additional explanatory variables alongside the model-derived seeding term. To examine potential stage-specific effects, we stratified the analysis by clinical diagnosis (CN, MCI and AD), fitting separate regression models within each group. We tested the null hypothesis that the slope of model-derived seeding is zero (two-sided Student’s *t*-test) with significance levels as defined for the simple regression. We used marginal effect plots to visualize the relationship between predictions and empirical tau while holding covariates at their mean values. Forest plots show the full set of standardized effect sizes, including covariates. Effect sizes were standardized for continuous, but not discrete, covariates.

## Results

**Figure 1:**
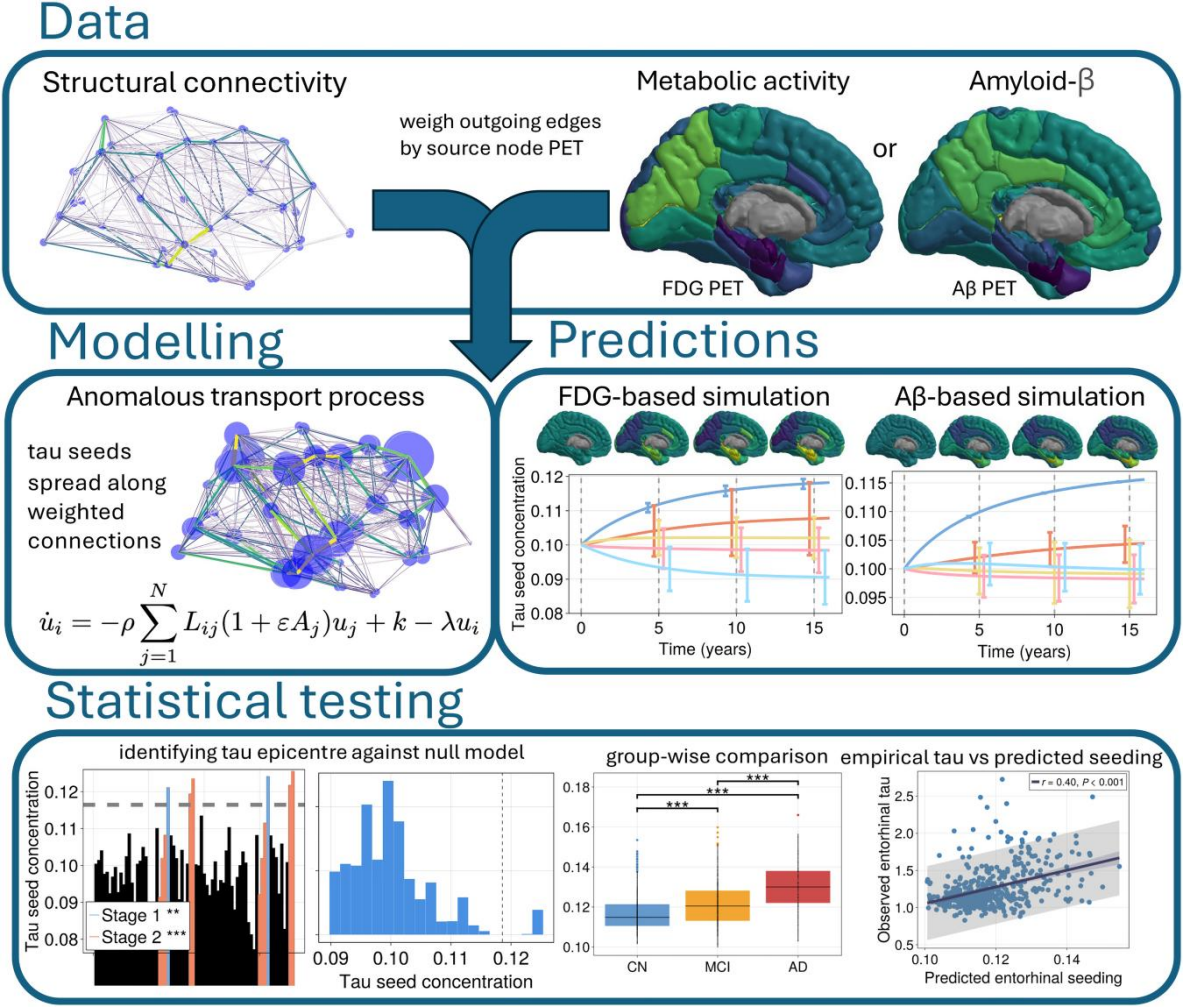
**Schematic overview of our framework for modelling tau seeding susceptibility.** We integrated structural connectivity with metabolic/neuronal activity (FDG PET) or Aβ deposition (Aβ PET), which are hypothesized to accelerate tau seed transport. Scaling the protein transport along structural connections by their regional PET SUVRs, we constructed a mathematical model to simulate and predict regional susceptibility to tau seeding. To evaluate our predictions, we conducted three statistical tests: (i) testing the significance of our seeding predictions by shuffling regional PET values, generating a null distribution where the spatial pattern of neuronal activity or amyloid deposition is randomized; (ii) assessing whether predicted seeding concentrations in the entorhinal cortex differ between subject groups stratified by diagnosis and biomarker status; and (iii) testing whether model-derived tau seeding correlates with empirical tau accumulation across subjects. Note: Some simulation panels are reused in later figures. Aβ = amyloid-β; AD = Alzheimer’s disease; CN = cognitively normal; FDG = fluorodeoxyglucose; MCI = mild cognitive impairment; SUVR = standardized uptake value ratio.

To investigate how neuronal activity and Aβ deposition shape regional susceptibility to tau seeding, we integrated empirical FDG PET and Aβ PET imaging data with a mathematical framework describing interneuronal tau transport. A schematic overview of our approach is shown in Fig. 1, outlining key steps in our modelling framework and analysis. In the following sections, we present our key findings: (i) brain-wide neuronal activity gradients predict tau seeding in the medial temporal lobe regardless of Aβ status and diagnosis; (ii) Aβ deposition selectively amplifies tau seeding in the entorhinal cortex; (iii) seeding predictions in early Braak stages are statistically significant upon shuffling PET SUVRs; and (iv) subject-level seeding predictions correlate with early empirical tau deposition.

### Metabolic activity drives the seeding of tau in the medial temporal lobe

Many elderly people exhibit tauopathy in the medial temporal lobe, which can occur in the absence of Aβ plaques, as in primary age-related tauopathy.^5^ Motivated by the hypothesis that neuronal activity influences where tau seeding begins, we used a computational model in which tau seeds spread along structural connections and is modulated by FDG PET activity. Group-averaged FDG PET distributions for each subject subgroup are visualized in Supplementary Fig. 1.

**Figure 2:**
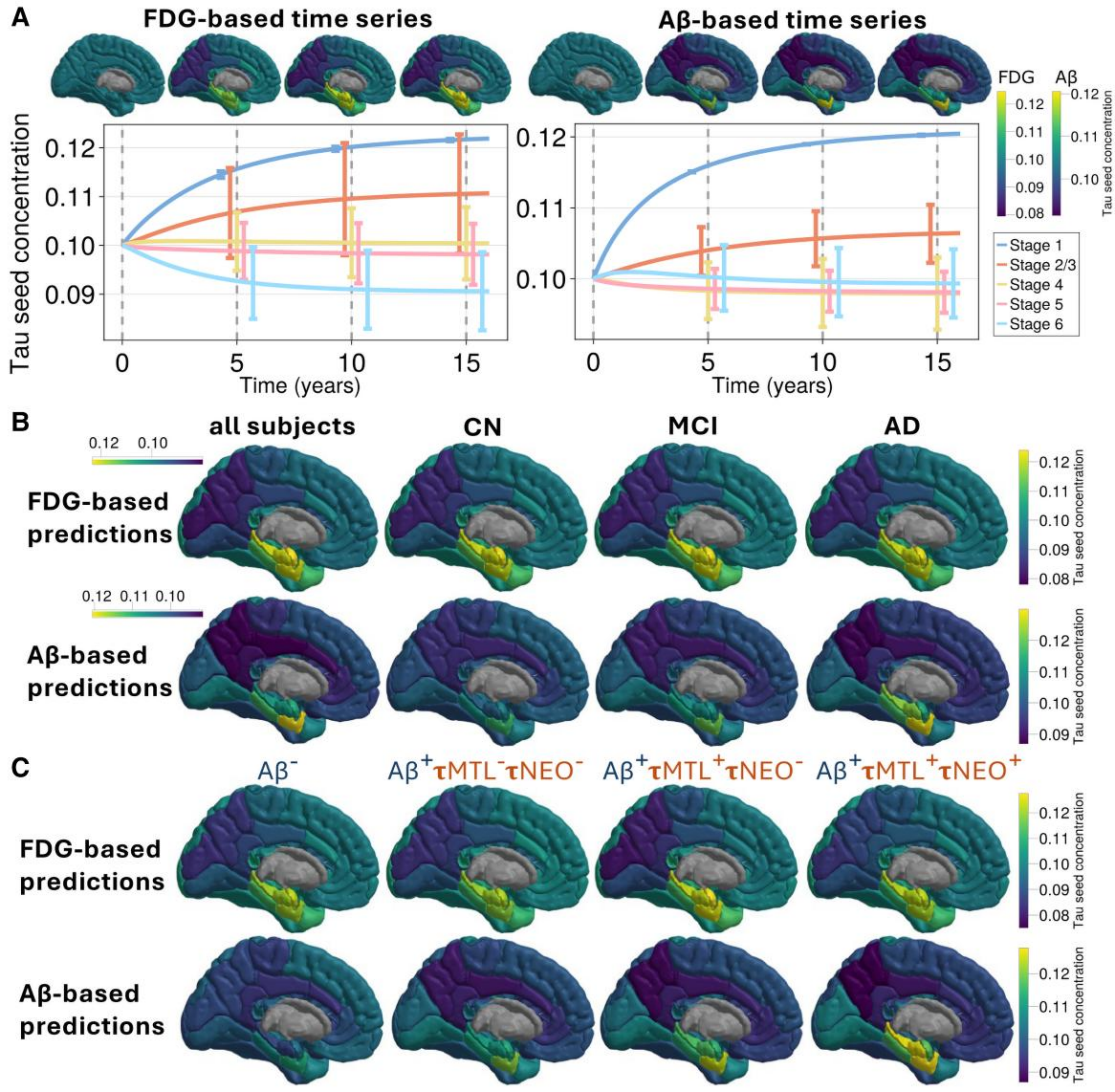
**Simulated tau seeding across time and subject groups.** (**A**) Temporal evolution of tau seed concentrations in a computational model informed by either FDG-PET or Aβ-PET. PET SUVR values were averaged across all subjects. The *top row* shows simulated tau seeding mapped onto 3D renderings of the left hemisphere, and the lower row displays the corresponding time series. Blue lines denote Braak stage 1 regions (entorhinal cortex); orange lines denote Braak stage 2/3. Time units are arbitrary and used illustratively; *T* = 0 reflects the model’s uniform initial condition. (**B**) Steady-state (asymptotic) tau seeding predicted by the model across clinical groups: CN (cognitively normal), MCI (mild cognitive impairment), AD (Alzheimer’s disease) and All Subjects. Columns represent group-averaged PET inputs (FDG *top*, Aβ *bottom*); model predictions were run independently per group. The diagnosis subgroups share a colour bar for ease of comparison, while the All Subjects group uses a separate one. (**C**) As in **B**, but with subject groups defined by biomarker status: Aβ^+/−^ (amyloid status), τMTL^+/−^ (tau in the medial temporal lobe), and τNEO^+/−^ (tau in the neocortex). All biomarker groups share the colour bar. The modelling parameters as described in the ‘Materials and methods’ section. Aβ = amyloid-β; FDG = fluorodeoxyglucose; SUVR = standardized uptake value ratio.

As shown in Fig. 2A and B, using the averaged FDG PET across all subjects, we observed that metabolic activity steers the tau seeds towards the medial temporal lobe in the model simulations. Specifically, the entorhinal cortex, the hippocampus and the amygdala have a higher concentration of tau seeds than other regions. This pattern was replicated in the HABS cohort, where FDG-based predictions also concentrated in the medial temporal lobe, particularly the entorhinal cortex (Supplementary Fig. 8A and B).

### Neuronal activity promotes tau seeding independently of amyloid-β and tau status

Next, we examined the steady-state (long-term) tau seeding distributions across diagnosis and biomarker groups. Whereas Fig. 2A illustrated how simulated tau seed concentrations evolve over time, Fig. 2B and C focus solely on the final distribution of tau seeds once equilibrium is reached. Each panel in Fig. 2B and C reflects a different group-averaged PET input, enabling us to evaluate how regional vulnerability varies across subject groups.

FDG-based predictions of tau seeding in the medial temporal lobe generalize across subject classifications and remain robust under variation in modelling parameters (see Fig. 2B and C and Supplementary Fig. 2, respectively). We repeated the seeding prediction analysis across Aβ/tau subgroups and clinical diagnoses. For each group, we used their averaged FDG PET signals to weight the transport of tau in the model simulations. The medial temporal lobe was consistently the most vulnerable to seeding across all subject groups.

**Figure 3:**
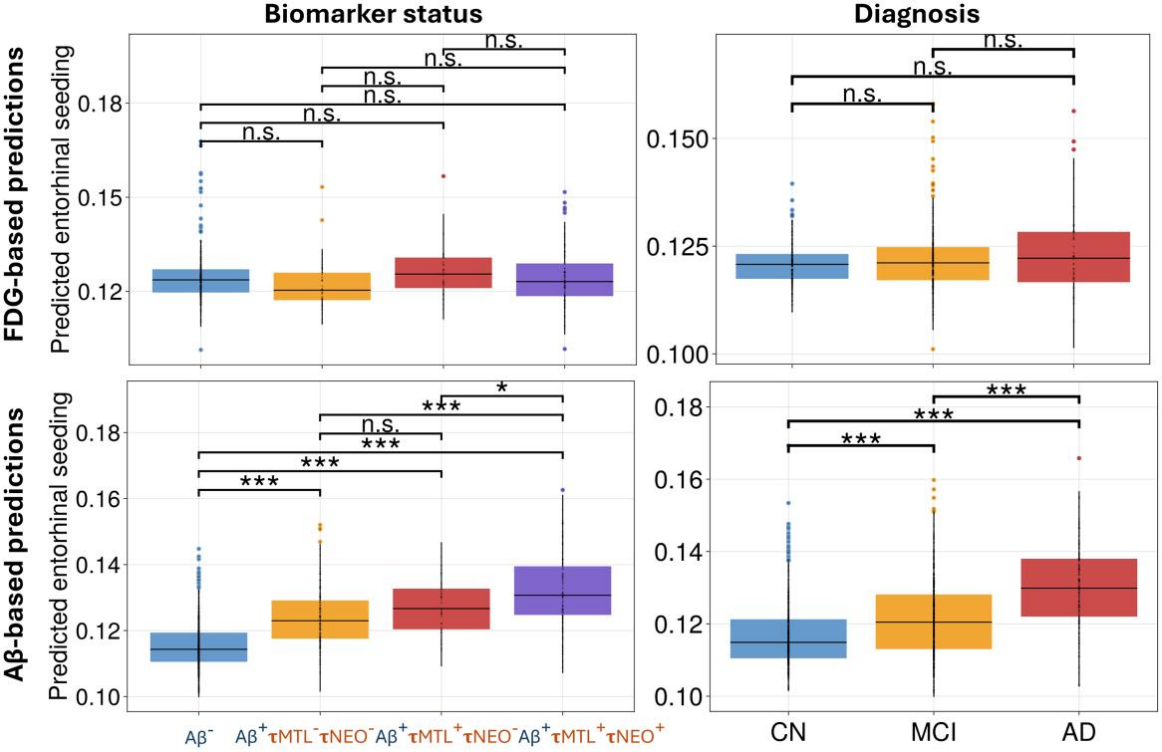
**Distributions of predicted entorhinal tau seeding values from subject-level simulations, stratified by diagnosis and biomarker classification**. The *top row* shows FDG-based predictions; the *second row* shows Aβ-based predictions. The *left column* displays classification based on amyloid and tau PET status, and the *right column* shows classification by clinical diagnosis. All panels show predicted seeding in the entorhinal cortex (Braak stage 1). Horizontal bars indicate pairwise comparisons between groups, with significance determined by two-sided Welch’s *t*-tests. Asterisks denote (corrected) statistical significance levels: *P < 0.05, **P < 0.01, ***P < 0.001, n.s. = not significant. Aβ = amyloid-β; FDG = fluorodeoxyglucose.

To further investigate the differences in tau seeding predictions across subject classifications, we computed the distributions in entorhinal seeding predictions in each classification. For each subject in each group, we incorporated their individual FDG PET images to make individualized seeding predictions. As shown in Fig. 3, there is little variation in entorhinal seeding concentrations across diagnosis and biomarker status. Pairwise Welch’s *t*-tests confirmed that entorhinal seeding estimates did not differ between any of the four FDG biomarker groups (|*t*| ≤ 2.36; smallest corrected *P* = 0.13; Supplementary Table 2). Likewise, when subjects were grouped by clinical diagnosis (CN, MCI and AD), predicted seeding distributions overlapped substantially and all pairwise contrasts were non-significant (|*t*| ≤ 1.67; smallest corrected *P* = 0.29).

### Aβ deposition drives tau seeding in the entorhinal cortex

The medial temporal lobe—in particular, the entorhinal cortex—is the first to develop tauopathy in AD, where, unlike primary age-related tauopathy, subjects also have Aβ pathology. Furthermore, research suggests that Aβ accelerates the spread of tau pathology,^21,22^ possibly through Aβ-induced hyperexcitability.^23-25^ If this is indeed the case, the presence of Aβ may increase the transport of tau seeds. For this reason, we repeated the simulations and analyses of the previous section, but where Aβ PET signals replace the FDG PET.

As shown in Fig. 2A and B, weighting the model with Aβ signal (averaged over all subjects) leads to a pronounced seeding prediction in the entorhinal cortex. While FDG-based weighting highlights multiple medial temporal regions—including the entorhinal cortex, hippocampus, and amygdala—Aβ weighting concentrates the effect specifically on the entorhinal cortex. Moreover, the disparity in seeding concentrations between the entorhinal cortex and the rest of the brain is greater for Aβ-based predictions than for FDG-based predictions, as seen in Fig. 2A. Replication in the HABS cohort showed that Aβ-based predictions again strongly localized to the entorhinal cortex (Supplementary Fig. 8A and B), consistent with the ADNI results.

### Amyloid-β-induced tau seeding differs across subject groups

The Aβ-based predictions show greater variation between subject groups (using the average Aβ PET signals from the different subject classifications to inform the model) than the FDG-based predictions. Perhaps unsurprisingly, the Aβ^−^ group exhibit the least tau seeding, similarly to the cognitively normal diagnosis group. In contrast, the Aβ-positive groups consistently show greater accumulation in the entorhinal cortex (Fig. 2C). This pattern remains consistent across different levels of *ɛ*, modulating the impact of Aβ on tau transport (Supplementary Fig. 3). For example, the Aβ^+^τMTL^+^τNEO^−^ group (exhibiting tau accumulation in the medial temporal lobe but not in the neocortex) shows a particularly strong bias towards tauopathy in the entorhinal cortex alone. Moreover, the Alzheimer’s diagnosis group presents a broader pattern that extends into the hippocampus, matching the Aβ^+^τMTL^+^τNEO^+^ classification. These findings suggest that early Aβ patterns are particularly conducive to tau seeding in the entorhinal cortex.

We performed two-sided Welch’s *t*-tests to evaluate group differences in model-derived tau seeding estimates within the entorhinal cortex. As shown in Fig. 3, both biomarker and diagnosis stratifications reveal a graded increase in predicted seeding across the AD continuum. In the Aβ biomarker groups, predicted entorhinal seeding increased steadily across Aβ⁻, Aβ⁺τMTL⁻τNEO⁻, Aβ⁺τMTL⁺τNEO⁻ and Aβ⁺τMTL⁺τNEO⁺, with all pairwise comparisons statistically significant (|*t*| ≥ 2.87, *P* < 0.029) except between the two intermediate Aβ⁺ subgroups (Aβ⁺τMTL⁻ τNEO⁻ versus Aβ⁺τMTL⁺τNEO⁻, *t* = −2.17, *P* = 0.20). Tau seeding predictions were higher in MCI than CN (*t* = −7.5, *P* < 0.001) and highest in AD (versus CN: *t* = −13.1; versus MCI: *t* = −8.7; all *P* < 0.001). These findings show that the predicted vulnerability of the entorhinal cortex increases with advancing disease severity across both clinical and biomarker-defined groups.

### Neuronal activity and amyloid-β are significant predictors of tau seeding in the medial temporal lobe

To test whether the model's seeding predictions are statistically significant, we constructed a null model by randomly shuffling PET signals across regions. For each simulation, we identified a null threshold and defined seeding regions as those exceeding it (see Supplementary Fig. 4 and the ‘Materials and methods’ section for details). We then tested whether the model predicts tau seeding in the entorhinal cortex or early Braak stages more often than expected by chance.

**Figure 4:**
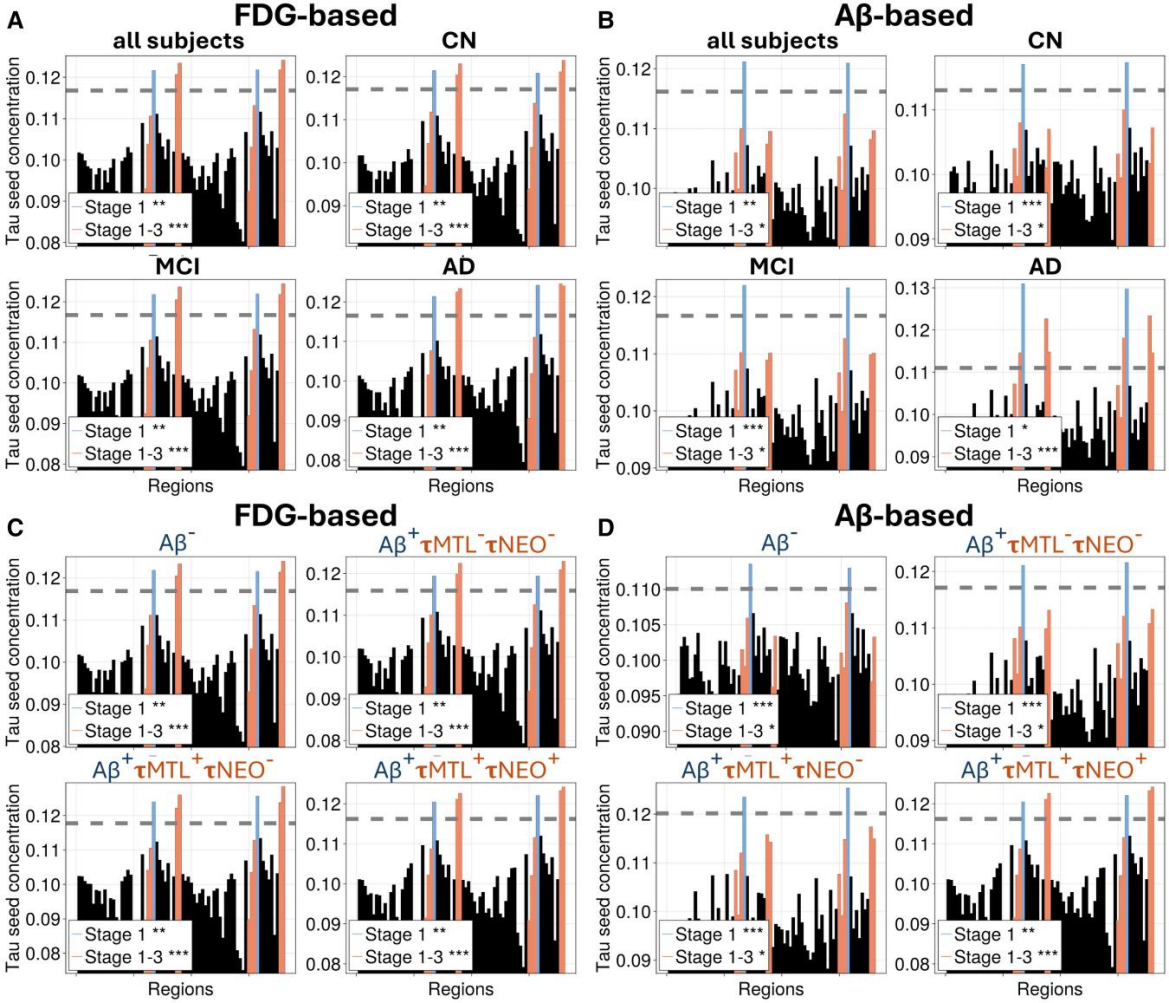
**Statistical testing of predicted tau seeding regions across subject groups.** Predicted regional tau seed concentrations from the FDG-informed (**A** and **C**) and Aβ-informed (**B** and **D**) models are shown for different diagnostic (**A** and **B**) and biomarker-defined (**C** and **D**) subgroups. Each bar plot displays predicted asymptotic (steady state) tau seeding concentrations across regions, with Braak stage 1 (entorhinal cortex) regions coloured in blue and Braak stage 2/3 regions in orange. The grey dashed line represents the seeding threshold determined by the null model (Supplementary Fig. 4). Asterisks indicate the significance level of null hypothesis testing: the probability of observing as many or more regions above threshold in Braak stage 1 or Braak stages 1–3 when regional PET SUVR values are randomly shuffled. *P < 0.05, **P < 0.01, ***P < 0.001. Modelling parameters are as described in the ‘Materials and methods’ section. Aβ = amyloid-β; AD = Alzheimer’s disease; CN = cognitively normal; FDG = fluorodeoxyglucose; MCI = mild cognitive impairment; SUVR = standardized uptake value ratio.

FDG- and Aβ-based models predicted tau seeding in Braak stage 1 (bilateral entorhinal cortex) and stages 1–3 significantly more often than expected by chance across all diagnostic and biomarker-defined subgroups (Fig. 4 and Supplementary Tables 3 and 4). For FDG-based predictions, Braak 1 *z*-scores ranged from 4.66 to 4.86 (*P* < 0.0066), and stage 1–3 *z*-scores ranged from 5.68 to 5.78 (*P* < 0.001). The Aβ-based model similarly showed strong effects across all groups with Braak 1 *z*-scores ranging from 4.05 to 8.66 (*P* < 0.012) and stage 1–3 *z*-scores from 3.14 to 6.69 (*P* < 0.027). Permutation tests in the HABS cohort confirmed statistically significant seeding in the entorhinal cortex for both FDG- and Aβ-based models (*P* = 0.030 and *P* = 0.0018, respectively; see Supplementary Fig. 8C and Supplementary Table 7).

Overall, across every stratification, the entorhinal cortex—and other early Braak regions—were identified by the model as a tau seeding region at rates far exceeding those expected under randomized conditions.

### Subject-level neuronal activity and amyloid deposition inform early tau accumulation

**Figure 5:**
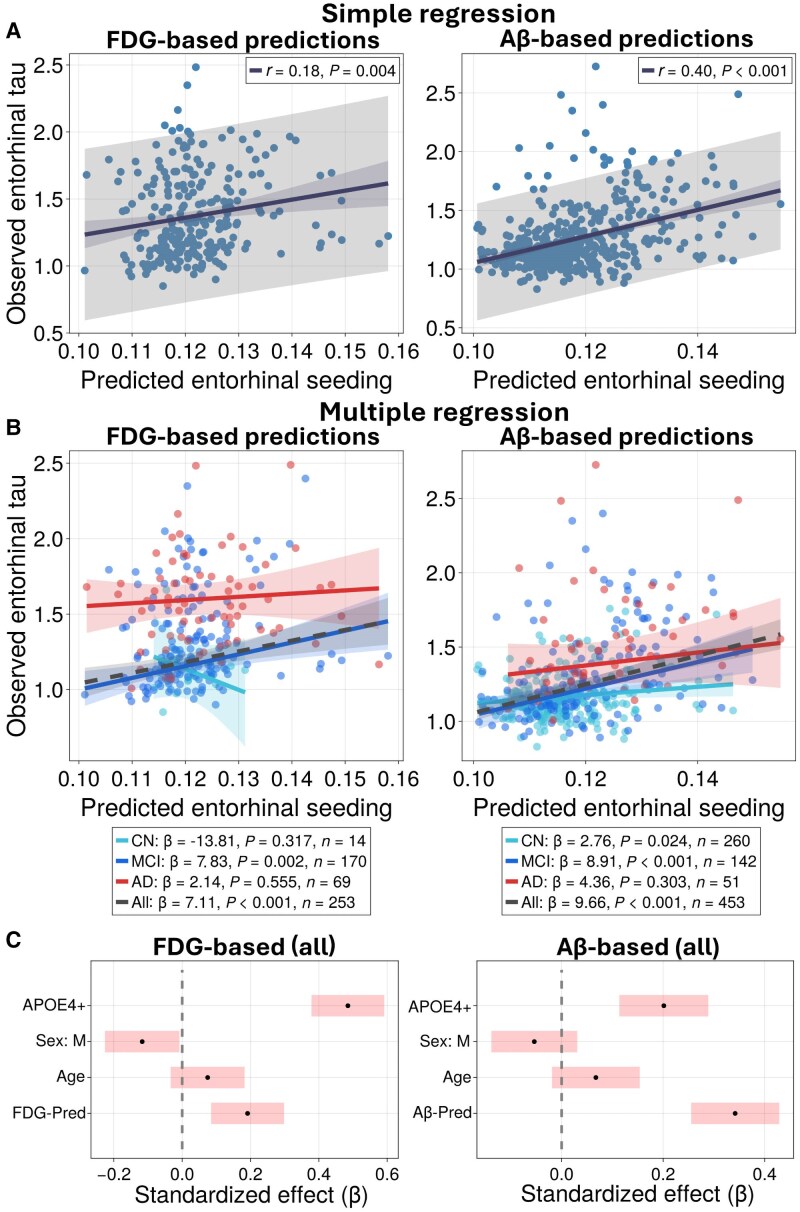
**Regression analysis linking model-predicted tau seeding with empirical tau PET in the entorhinal cortex.** (**A**) Simple linear regression between model-derived seeding and empirical tau PET SUVRs in the entorhinal cortex across all subjects. FDG- (*left*) and Aβ-based (*right*) predictions. Dots represent subjects; lines show least-squares fits with 95% confidence (red) and prediction (grey) intervals. Pearson *r* and *P*-values are reported. (**B**) Marginal effect plots from multiple linear regressions adjusted for age, sex and APOE4, stratified by diagnosis. Lines show predicted entorhinal tau as a function of model-derived seeding, with covariates held at their mean. Reported statistics include slope (raw *β*), *P*-value and *n*. (**C**) Standardized effect sizes with 95% confidence intervals fitted across all subjects. Dots indicate point estimates; bars indicate confidence intervals. Modelling parameters are as described in the ‘Materials and methods’ section. Aβ = amyloid-β; Aβ = amyloid-β; AD = Alzheimer’s disease; CN = cognitively normal; FDG = fluorodeoxyglucose; MCI = mild cognitive impairment; SUVR = standardized uptake value ratio.

In the previous section, we used group-averaged PET scans to examine how FDG and Aβ patterns influence early tau seeding. Here, we ask whether individual seeding predictions correlate with tau levels in the entorhinal cortex. A simple linear regression showed a weak but reliable correlation for FDG [*r* = 0.18, unstandardized *β* = 6.71, 95% confidence interval (CI) (2.17, 11.25), *P* = 0.0039, *n* = 253; see Supplementary Table 5 for detailed statistics] and a moderate correlation for Aβ [*r* = 0.40, unstandardized *β* = 11.26, 95% CI (8.86, 13.67), *P* < 0.001, *n* = 453 as seen in Fig. 5A]. Correlations were also found for Braak stage 2/3 regions in the Aβ-based model (*r* = 0.40, *P* < 0.001, see Supplementary Fig. 7A).

After adjusting for age, sex, and APOE4 status, the model-derived seeding term remained significant across all subjects for both FDG- (standardized *β* = 0.19, unstandardized *β* = 7.1, *P* < 0.001) and Aβ-based predictions (standardized *β* = 0.34, unstandardized *β* = 9.7, *P* < 0.001) as seen in Fig. 5B and C. Model fit (predicted versus observed and quantile-quantile plots) and summary statistics are found in Supplementary Fig. 6 and Supplementary Table 6, respectively. Regression within each diagnosis group revealed a significant FDG-based effect for MCI (standardized *β* = 0.21, unstandardized *β* = 7.8, *P* = 0.002, *n* = 170), and significant Aβ-based effects for CN (standardized *β* = 0.14, unstandardized *β* = 2.8, *P* = 0.024, *n* = 260) and MCI (standardized *β* = 0.30, unstandardized *β* = 8.9, *P* < 0.001, *n* = 142), as seen in Fig. 5B. Standardized covariate effects with confidence intervals for the diagnosis subgroups are shown in Supplementary Fig. 5. For Braak stages 2/3, correlations were also found for the Aβ-based model after covariate adjustment (standardized *β* = 0.36, *P* < 0.001; Supplementary Fig. 7B and C).

In HABS, Aβ-based subject-level predictions significantly correlated with empirical entorhinal tau both in simple (*r* = 0.29, *P* < 0.001) and multiple regression (standardized *β* = 0.17, *P* = 0.0088), while FDG-based predictions did not reach significance—possibly due to reduced variability and limited statistical power (Supplementary Figs 8D, 11–15 and Supplementary Tables 8 and 9).

These findings lend support to the hypothesis that FDG and Aβ patterns play a role in early tau seeding, as the predicted seeding aligns with empirical tau deposition across subjects.

## Discussion

The medial temporal lobe—particularly the entorhinal cortex—is consistently the first region to develop tau pathology in both primary age-related tauopathy and AD. However, the underlying reasons for its heightened susceptibility to tau seeding remain unclear. Here, we demonstrate that activity-dependent protein transport predicts tau seeding in the medial temporal lobe. Our results support the hypothesis that neuronal activity promotes tau seeding in this region, helping explain its vulnerability in primary age-related tauopathy. Assuming that Aβ accelerates tau spread, we further show that brain-wide Aβ patterns contribute an even greater vulnerability to the entorhinal cortex, consistent with early Alzheimer’s pathology. These results suggest that brain-wide patterns of neuronal activity and amyloid deposition may be the underlying force driving tau seeding in primary-age-related tauopathy and AD.

### Contextualizing medial temporal lobe seeding within whole brain network changes

Recently, Giorgio *et al.*^46^ showed with task-based functional MRI (fMRI) that Aβ deposition in the default mode network induces a switch from inhibition to activation of the medial temporal lobe and that this directed activation of the medial temporal lobe correlates with tau accumulation. This result corroborates the hypothesis that Aβ-induced hyperactivity leads to tau accumulation in the medial temporal lobe, which is also supported by our findings. Moreover, our results provide a mechanism for the Aβ-induced tau accumulation, where the Aβ-induced hyperexcitability in the default-mode network increases the transport of tau seeds into the medial temporal lobe. Another recent study by Hojjati *et al.*^47^ demonstrated that Aβ deposition is correlated with increased inter-network resting-state fMRI functional connectivity in the limbic, default mode, and control network. Moreover, increases in functional connectivity between the default-mode and limbic networks correlated with increases in tau PET SUVRs in the limbic network in Aβ-positive, healthy control individuals but not in Aβ-negative, healthy control individuals.^47^ Furthermore, Roemer-Cassiano *et al.*^48^ found higher tau accumulation in regions exhibiting Aβ-induced increases in resting-state fMRI functional connectivity to tau epicentres. These findings suggest that Aβ asserts its impact on tau spreading by modulating neuronal communication between brain regions. However, it is unclear how activity-dependent tau transport relates to fMRI functional connectivity metrics, as these measure a notion of synchronicity in neuronal activity between regions and not general neuronal activity levels. These studies establish that functional connectivity changes induced by Aβ may initiate the spread from the medial temporal lobe to the limbic network and the neocortex. In our study, in contrast, we provide support for the hypothesis that neuronal activity steers tau seeds into the medial temporal lobe and that Aβ may exacerbate the particular susceptibility of the entorhinal cortex. Although Aβ deposition is necessary for Alzheimer-like propagation of tau in the neocortex, it is not necessary for tau accumulation in the medial temporal lobe, as observed in primary age-related tauopathy. Considering these recent findings^46-48^ alongside our own, we propose the hypothesis that: (i) brain-wide neuronal activity promotes the accumulation of tau seeds in the medial temporal lobe regardless of Aβ status; and (ii) Aβ-induced hyperexcitability leads to an even higher accumulation in the entorhinal cortex, crossing a critical threshold for which tau starts to spread to the limbic network and eventually the neocortex.

While our results indicate that there may be remote effects of neuronal activity and Aβ that promote the vulnerability of the entorhinal cortex to tau accumulation in AD, other potential mechanisms for the early involvement of entorhinal cortex pathology also exist. For example, studies have shown that hypoperfusion in the entorhinal cortex precedes tau accumulation,^49^ and that the cellular makeup of the entorhinal cortex may predispose it to tau accumulation.^50^ As yet, it is unclear how different regional vulnerabilities may contribute to the ultimate manifestation of initial tau pathology in the entorhinal cortex.

In this context, our model raises the hypothesis that elevated neuronal activity in the medial temporal lobe, such as in the hippocampus, may facilitate outward redistribution of tau seeds, thereby limiting local accumulation. This framework might help interpret findings that individuals engaged in spatially demanding professions, such as taxi drivers,^51^ are less likely to die from AD, which may be related to structural changes in the hippocampus.^52^ However, the dynamics of hippocampal activity in AD are complex, with some studies associating hyperactivity with tau pathology in the hippocampus.^53,54^ While our model assumes that activity promotes outward tau transport, this is likely an oversimplification; neuronal activity may in some cases promote clearance, while in others enhance seeding. Nonetheless, recent work suggests that stimulation of medial temporal lobe regions, such as the entorhinal cortex, can reduce tau levels and improve cognitive function in mouse models,^55,56^ potentially by engaging activity-dependent transport mechanisms.

### Methodological considerations

While FDG PET provides a robust measure of neuronal activity, it does not capture the full complexity of the neurophysiological processes that may influence tau propagation, such as synaptic activity or intracellular signalling pathways and may be complemented with fMRI, MEG, EEG and transcriptomic data in future studies. We have also assumed that neuronal activity remains unchanged through time, whilst neuronal activity itself is a dynamical process evolving over time. Previous models have investigated the co-evolving dynamics of neuronal activity and protein spreading and may be used forward to test further hypotheses on the activity-spreading link.^29,57^

Our results are limited by the lack of directedness in structural connectivity, for which no consensus inference method exists for human imaging. Without directionality in the connectome edges, the structural connectivity does not impact the steady-state tau accumulation in each region, though it does impact the temporal spreading patterns. We use a structural connectome derived from young, healthy individuals in the Human Connectome Project, as appropriate for modelling the earliest stages of disease. Our model is designed to capture how tau pathology may first emerge and become regionally biased, assuming intact anatomical connectivity. In future extensions, the framework could be expanded to incorporate progressive structural degeneration as a downstream consequence of tau accumulation, allowing the connectome to evolve over time and potentially influence later-stage propagation dynamics.^57-59^ Nonetheless, our results show that the effect of including the anomalous transport process (neuronal activity and Aβ) biases the accumulation of tau towards the medial temporal lobe and entorhinal cortex, regardless of the topology of the structural connectome. This is because the asymptotic tau concentration in each region *i* scales with 1/(1 + *ɛA*_i_) independently of the underlying structural connectivity (see the ‘Materials and methods’, ‘Computational modelling’ section). While our model suggests that regional PET gradients shape the spatial distribution of tau seeding, a natural next step is to better understand which regions or pathways contribute to entorhinal vulnerability. With directed structural connectivity, this would be analytically tractable by investigating the dominant eigenvector of the activity-scaled asymmetric Laplacian. However, in the current undirected setting, no single region can be isolated as the ‘source' of entorhinal tau, since spreading is governed by symmetric flows. Still, the topology influences the dynamics, and future studies could analyse early propagation patterns by examining the second and third eigenvectors of the structural (symmetric) Laplacian, which represent slow diffusion modes and have been used in prior work to infer dominant spreading pathways.^41^ While beyond the scope of this study, such extensions could help pinpoint network routes most responsible for early-stage tau accumulation.

Moreover, while our null model tests for region-specific seeding predictions by randomizing PET values across ROIs, future work could incorporate spatial autocorrelation–preserving approaches such as the spin test,^60^ which may provide additional insight into spatial correspondence between predicted and empirical patterns. Our model also assumes a linear relationship between neuronal activity, Aβ, and tau transport. However, the interaction between these elements may be non-linear or context dependent. Furthermore, it is possible to expand our computational model to include non-linear effects mimicking regional prion-like replication.^61^ Such a model can then simulate the initiation of the disease in the entorhinal cortex in addition to the ensuing spatiotemporal Braak staging pattern without the need to artificially instruct the computational model where to begin tau pathology. As such, computational modelling of prion-like spreading may not only predict individual spreading patterns but also identify the brain regions where propagation is most likely to initiate. Indeed, it has been established that there is considerable variation in the localization of tau pathology between individuals with AD,^62^ raising the question whether this variability may be, in part, accounted for by differences in brain-wide neuronal activity and Aβ deposition.

### Replication in an independent cohort

To assess the robustness of our findings, we applied the same modelling framework to an independent cohort from the Harvard Aging Brain Study. Both the FDG- and Aβ-based models again identified the entorhinal cortex as a vulnerable target of early tau seeding, with results reaching significance under a permutation-based null model. In contrast to the ADNI cohort, the pars orbitalis was also significantly identified as a tau seeding region for Aβ-based predictions, in line with reports of early tau PET signal in this region (possibly confounded by off-target tracer binding) and evidence of age-related vulnerability.^63,64^ At the individual subject level, Aβ-based predictions significantly correlated with entorhinal tau-PET signal, replicating the main result from ADNI and reinforcing the role of spatial Aβ gradients in shaping early tau vulnerability. The FDG-based correlation did not reach significance in HABS. This may reflect a combination of factors, including limited statistical power (our resampling analysis estimated only 49% power at the available sample size; Supplementary Fig. 14), reduced variability in entorhinal tau signal relative to ADNI (Supplementary Fig. 15), and differences in cohort composition, as HABS consists primarily of cognitively normal individuals while ADNI includes a larger proportion of participants with mild cognitive impairment. While the subject-level FDG-based correlation did not reach significance in HABS, the model’s consistent identification of the entorhinal cortex as a primary seed region across both cohorts supports the relevance of PET-derived spatial gradients. The differences between cohorts also underscore the need to account for variability in disease stage and tau signal when interpreting subject-level effects.

## Conclusion

We showed that brain-wide patterns of neuronal activity and Aβ may underlie the vulnerability of the entorhinal cortex to tau seeding. Our findings suggest that neuronal activity—irrespective of Aβ—biases tau seeding toward the medial temporal lobe, consistent with primary age-related tauopathy. Moreover, our model indicates that Aβ amplifies entorhinal vulnerability, potentially pushing the region past a critical threshold initiating Alzheimer’s-like pathology. Future work should investigate how spatial gradients of neuronal activity and Aβ jointly shape early tau vulnerability, and whether modulation of these gradients could help mitigate the earliest stages of disease.

## Supplementary Material

awaf374_Supplementary_Data

## Data Availability

Code is available upon request.
