# Supplementary material for: Neuronal activity and amyloid-β promote tau seeding in the entorhinal cortex in Alzheimer’s disease
Source: Brain. 2025 Oct 7;149(6):1915–28. doi: 10.1093/brain/awaf374 (PMC13233043; doi:10.1093/brain/awaf374)
Supplement: awaf374_Supplementary_Data [file awaf374_supplementary_data.pdf]

Supporting information for

Neuronal activity and amyloid-β promote tau seeding in the entorhinal cortex in Alzheimer's disease

Christoffer G. Alexandersen[1,2], Dani S. Bassett[1,3,4,5,6,7], Alain Goriely[2,✉], Pavanjit Chaggar[2,8], and the Alzheimer's Disease Neuroimaging Initiative *

[1]Department of Bioengineering, School of Engineering and Applied Science, University of Pennsylvania

[2]Mathematical Institute, University of Oxford

[3]Department of Electrical & Systems Engineering, School of Engineering and Applied Science, University of Pennsylvania

[4]Department of Physics & Astronomy, School of Arts & Sciences, University of Pennsylvania

[5]Departments of Neurology & Psychiatry, Perelman School of Medicine University of Pennsylvania

[6]The Neuro, Montreal Neurological Institute, McGill University

[7]Santa Fe Institute

[8]Clinical Memory Research, Lund University

PET data processing

We use PET data from the Alzheimer's Disease Neuroimaging Initiative (ADNI, adni.loni.usc.edu). ADNI is a public-private partnership with the aim of using serial biomarkers to measure the progression of Alzheimer's disease. For up-to-date information, see www.adni-info.org. For Aβ (florbetapir PET) and tau (flortaucipir PET), we download the fully processed PET tabular data, summarized as standardized uptake value ratios (SUVR) on the Desikan-Killiany atlas.[30] Subjects included in the analysis each contribute a single Aβ and tau PET scan. We stratify the scans by diagnosis and biomarker status (Aβ and tau status). To group each subject by diagnosis, we identify their earliest available Aβ PET scan and use the diagnosis at the time of that scan. If no diagnosis is available, the scan is excluded. This yields 501 cognitively normal (CN), 588 mildly cognitively impaired (MCI), and 155 Alzheimer's disease (AD) scans. As for the biomarker stratification, the earliest available pair of Aβ and tau PET scans taken within ± 12 months was used to classify participants by Aβ and tau status. Subjects without eligible scan pairs are excluded, but no exclusion is made based on available diagnosis. Aβ SUVR was calculated using the whole cerebellum as reference region; then, Aβ status was classified as Aβ-positive (Aβ^+) if the ADNI cortical summary region SUVR (comprising the bilateral frontal, anterior/posterior cingulate, lateral parietal, lateral temporal regions) > 1.11 (20 CL) and Aβ-negative (Aβ^-) otherwise.[31] Tau PET SUVR was calculated using the inferior cerebellar as a reference region. Tau status was stratified into two categories based on the SUVR in two composite regions: a medial temporal lobe (τMTL$^{+/-}$) composite, comprising the bilateral entorhinal cortices and amygdalae, and a neocortical area (τNEO$^{+/-}$), comprising the bilateral inferior and middle lateral temporal lobes. The threshold for tau composite SUVR using an inferior cerebellum reference are 1.375 and 1.395 for the MTL and neocortical composites, respectively, and are derived using regional Gaussian mixture modeling (see Chaggar et al.[32]

*Data used in preparation of this article were obtained from the Alzheimer's Disease Neuroimaging Initiative (ADNI) database (adni.loni.usc.edu). As such, the investigators within the ADNI contributed to the design and implementation of ADNI and/or provided data but did not participate in the analysis or writing of this `http://adni.loni.usc.edu/wp-content/uploads/how_to_apply/ADNI_Acknowledgement_List.pdf`

✉goriely@maths.ox.ac.uk

for details). Partial-volume correction was not applied. We consider the following biomarker groups: Aβ^- (amyloid-negative), Aβ^+ τMTL$^-\tau$NEO$^-$(amyloid-positive, tau-negative in medial temporal lobe, tau-negative in neocortex), Aβ^+ τMTL$^+\tau$NEO$^-$(amyloid-positive, tau-positive in medial temporal lobe, tau-negative in neocortex), and Aβ^+ τMTL$^+\tau$NEO$^+$(amyloid-positive, tau-positive in medial temporal lobe, tau-positive in neocortex). For Aβ PET scans, we extracted 686 scans in Aβ^-, 131 scans in Aβ^+ τMTL$^-\tau$NEO$^-$, 43 scans in Aβ^+ τMTL$^+\tau$NEO$^-$, and 84 scans in Aβ^+ τMTL$^+\tau$NEO$^+$. We exclude the Aβ^+ τMTL$^-\tau$NEO$^+$group because it contained only 10 participants. The Aβ^- group was not filtered by tau status and contains 20 amyloid-negative tau-positive and 666 amyloid-negative tau-negative scans. Demographic characteristics of the Aβ PET study participants are summarized in Table 1.

FDG-PET scans were downloaded from ADNI in their maximally preprocessed form, which includes frame averaging and smoothing with a scanner-specific 6mm full-width at half-maximum (FWHM) Gaussian filter. The raw closest-in-time MRI image was also downloaded for each subject. The PET and MRI images were then skull-stripped, and the MRI image was coregistered to the MNI152 template image. The subject-specific MRI-to-template-space transformation was applied to the subject's MRI-coregistered PET image to warp the PET image to the MNI152 template space. All coregistration was performed using the Symmetric Normalization (SyN) algorithm implemented in Advanced Normalization Tools (ANTs). Regional signal values were extracted in MNI space using binary masks corresponding to the Desikan–Killiany atlas, which were defined in template space and obtained by applying FreeSurfer segmentation and parcellation to the MNI152 template. The SUVR was calculated by dividing the mean FDG PET signal in each cortical ROI by the mean signal in the pons, used as the reference region, which is minimally affected by Alzheimer's disease.[33,34] Partial-volume correction was not applied. Subjects included in the analysis each contribute a single FDG PET scan and are stratified by diagnosis and biomarker status. To group participants by diagnosis, we identify their earliest available FDG PET scan and use their diagnosis at the time of the scan, giving 174 CN, 293 MCI, and 60 AD scans (scans without available diagnosis are excluded). To classify FDG scans based on their Aβ and tau status, we collect a subject's earliest FDG scan taken within $\pm$12 months of an Aβ and tau PET scan pair; subjects without eligible scan pairs are excluded, but no exclusion is made based on available diagnosis. We extracted 285 FDG PET scans in Aβ^-, 47 in Aβ^+ τMTL$^-\tau$NEO$^-$, 37 in Aβ^+ τMTL$^+\tau$NEO$^-$, and 88 in Aβ^+ τMTL$^+\tau$NEO$^+$. We exclude the Aβ^+ τMTL$^-\tau$NEO$^+$group because it contained only seven participants. The Aβ^- group was not filtered by tau status and contains 13 Aβ-negative tau-positive and 272 Aβ-negative tau-negative scans. Demographic characteristics of the FDG PET study participants are summarized in Table 1.

To address known signal instability in the frontal pole, temporal pole, and banks of the superior temporal sulcus—regions often affected by poor segmentation—we followed prior work by replacing the SUVR for each of these with the average of itself and its adjacent cortical ROIs (see Table S1).[35] This correction was applied uniformly across both FDG and amyloid PET modalities.

Regional SUVR adjustments due to signal instability

To mitigate signal instability in cortical regions prone to segmentation errors, we applied a correction procedure in which each affected region was replaced by the average of itself and its anatomically adjacent cortical ROIs, bilaterally. Table S1 lists the specific substitutions used for this correction.

Table S1: Regions corrected and their substitution neighbors (applied bilaterally).

**Corrected Region**	**Neighboring ROIs Averaged**
bankssts	inferiorparietal, middletemporal, superiortemporal
frontalpole	lateralorbitofrontal, medialorbitofrontal, rostralmiddlefrontal, superiorfrontal
temporalpole	inferiortemporal, middletemporal, superiortemporal, entorhinal

FDG and amyloid PET data

To assist with interpretation of the model predictions across subject groups, we visualized the group-averaged PET inputs (from ADNI) used to drive the simulations in Supplementary Figure S1. These include FDG-PET and florbetapir PET, averaged across subjects grouped either by clinical diagnosis

or by biomarker-defined categories. The PET data reflect the spatial distributions of metabolic or amyloid burden entered into the tau seeding model. Each row in the figure shows surface renderings of left-hemisphere cortical SUVRs, using a shared color scale within each PET modality and participant stratification (diagnoses and biomarker status).

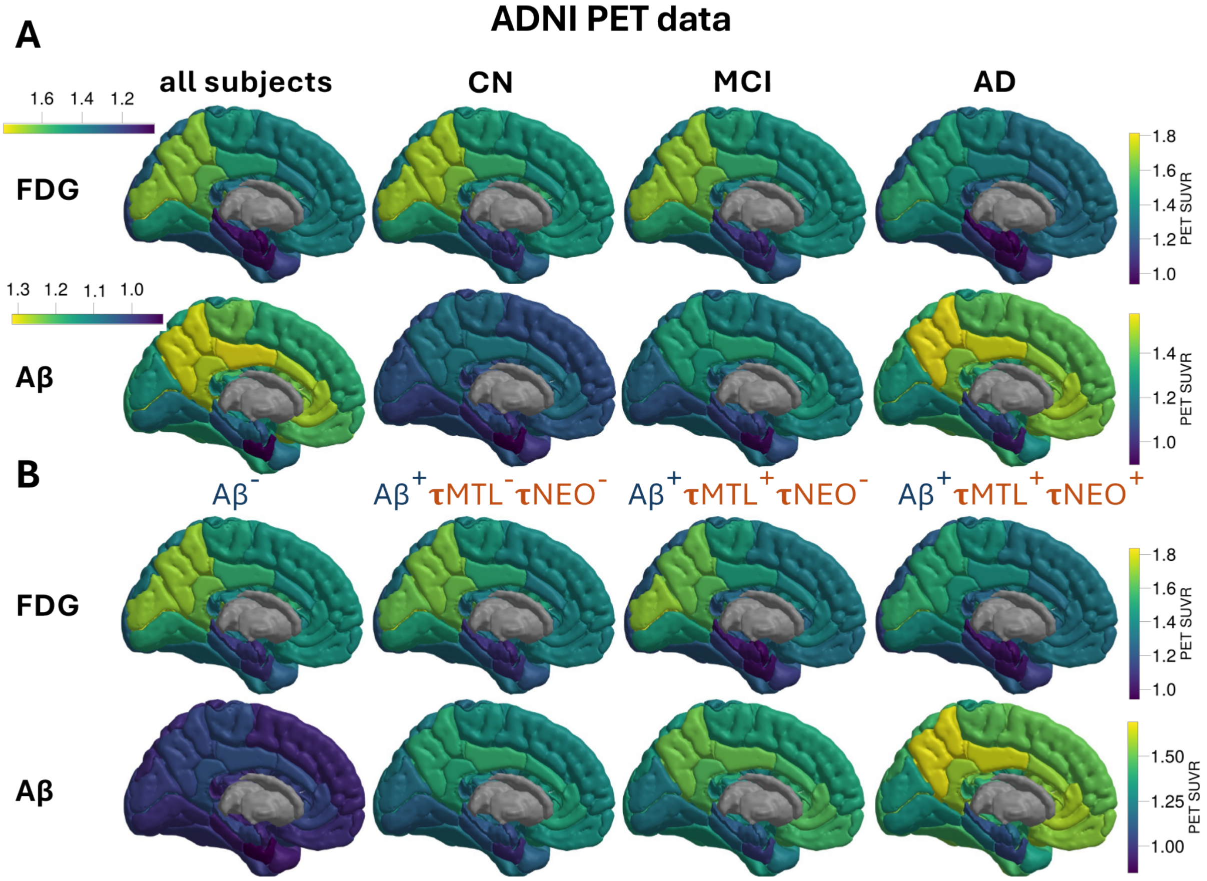

Figure S1: **Group-averaged PET SUVRs used as model input (ADNI).** **(A)** FDG-PET and amyloid-β PET SUVR maps averaged across subjects grouped by clinical diagnosis: cognitively normal (CN), mild cognitive impairment (MCI), Alzheimer's disease (AD), and all subjects. **(B)** PET SUVR maps for the same modalities, grouped by biomarker classification. Each map shows the average left-hemisphere cortical SUVRs projected onto a common surface.

Varying the impact of neuronal activity and amyloid on modeling predictions

By varying the control parameter ε, we adjust the impact that neuronal activity and amyloid-β has on the predicted seeding susceptibility across the brain. As seen in Figures S2 and S3, for low levels of ε there is no bias in seeding towards any particular region, and as ε is increased these biases become more apparent.

Group-level comparison of model predictions

Table S2 summarizes the results of pairwise Welch's t-tests comparing predicted tau seeding concentrations in the entorhinal cortex across subject groups. Tests were conducted separately for FDG- and Aβ-based PET classification/diagnosis groups.

Table S2: **Statistics for model prediction group differences (ADNI).** Pairwise Welch's t-tests for predicted tau seeding concentrations in the entorhinal cortex with Bonferroni-corrected p-values. Comparisons are grouped by FDG/amyloid PET classification and clinical diagnosis.

Grouping	Comparison	t-statistic	p-value (corr.)
FDG Biomarker	$A\beta^-$ vs $A\beta^+$ $\tau MTL^-\tau NEO^-$	2.04	0.28
FDG Biomarker	$A\beta^-$ vs $A\beta^+$ $\tau MTL^+\tau NEO^-$	-1.15	1.0
FDG Biomarker	$A\beta^-$ vs $A\beta^+$ $\tau MTL^+\tau NEO^+$	0.19	1.0
FDG Biomarker	$A\beta^+$ $\tau MTL^-\tau NEO^-$vs $A\beta^+$ $\tau MTL^+\tau NEO^-$	-2.36	0.13
FDG Biomarker	$A\beta^+$ $\tau MTL^-\tau NEO^-$vs $A\beta^+$ $\tau MTL^+\tau NEO^+$	-1.53	0.77
FDG Biomarker	$A\beta^+$ $\tau MTL^+\tau NEO^-$vs $A\beta^+$ $\tau MTL^+\tau NEO^+$	1.11	1.0
$A\beta$ Biomarker	$A\beta^-$ vs $A\beta^+$ $\tau MTL^-\tau NEO^-$	-10.19	< 0.001
$A\beta$ Biomarker	$A\beta^-$ vs $A\beta^+$ $\tau MTL^+\tau NEO^-$	-9.13	< 0.001
$A\beta$ Biomarker	$A\beta^-$ vs $A\beta^+$ $\tau MTL^+\tau NEO^+$	-13.31	< 0.001
$A\beta$ Biomarker	$A\beta^+$ $\tau MTL^-\tau NEO^-$vs $A\beta^+$ $\tau MTL^+\tau NEO^-$	-2.17	0.20
$A\beta$ Biomarker	$A\beta^+$ $\tau MTL^-\tau NEO^-$vs $A\beta^+$ $\tau MTL^+\tau NEO^+$	-5.65	< 0.001
$A\beta$ Biomarker	$A\beta^+$ $\tau MTL^+\tau NEO^-$vs $A\beta^+$ $\tau MTL^+\tau NEO^+$	-2.87	0.029
FDG Diagnosis	CN vs MCI	-1.40	0.48
FDG Diagnosis	CN vs AD	-1.67	0.29
FDG Diagnosis	MCI vs AD	-1.082	0.85
$A\beta$ Diagnosis	CN vs MCI	-7.49	< 0.001
$A\beta$ Diagnosis	CN vs AD	-13.07	< 0.001
$A\beta$ Diagnosis	MCI vs AD	-8.65	< 0.001

Seeding region prediction null models and statistics

Here we report null distributions and summary statistics for regional seeding predictions in both ADNI and HABS. For each group, we compare the number of regions exceeding the seeding threshold to the null expectation, stratified by diagnosis and biomarker status.

Null model for seeding susceptibility

As described in the Materials and methods section, we construct a null model for seeding predictions by randomly shuffling the PET SUVR values across brain regions in the model. This procedure disrupts any spatial specificity while preserving the global SUVR distribution. For each shuffle, we compute the asymptotic tau concentrations across all regions, pooling these values to form null distributions of predicted seeding concentrations. Figure S4 shows the resulting distributions for both FDG- and $A\beta$-based models, stratified by diagnostic and biomarker-defined groups. In each case, the grey stippled line denotes the seeding threshold, defined as the midpoint of the largest gap in the distribution above the median value.

Statistical validation of tau seeding predictions

To assess the significance of model-derived seeding predictions, we compared each result to a null distribution generated by 10,000 random spatial shuffles of the PET SUVR values across regions. A brain region was classified as a "seeding region" if its predicted asymptotic concentration exceeded the null-derived threshold (ADNI, see Fig. S4; HABS, see Fig. S10). We then quantified the frequency of seeding in Braak stage 1 (entorhinal cortex) and in early Braak stages 1–3. Tables S3 and S4 summarize the observed z-scores and corresponding p-values for each subgroup in ADNI.

Regression analysis

Here we summarize regression results comparing model-predicted tau burden to empirical tau PET across Braak-stage ROIs for the ADNI dataset.

Table S3: **Summary of statistical results for FDG-based model seeding site predictions (ADNI).** Each column corresponds to a subject group, and each row reports the observed number of seeded regions, null distribution mean and standard deviation, z-score, and p-value for Braak stage 1 (entorhinal cortex) and stages 1–3.

Measure	CN	MCI	AD	All	Aβ^-	Aβ^+ τMTL$^-$ τNEO$^-$	Aβ^+ τMTL$^+$ τNEO$^-$	Aβ^+ τMTL$^+$ τNEO$^+$
Stage 1 obs	2	2	2	2	2	2	2	2
Stage 1 null μ	0.166	0.159	0.168	0.166	0.162	0.170	0.168	0.168
Stage 1 null σ	0.387	0.379	0.39	0.386	0.382	0.392	0.392	0.390
Stage 1 z	4.743	4.862	4.702	4.754	4.810	4.664	4.677	4.701
Stage 1 p	0.0055	0.0049	0.0058	0.0053	0.0053	0.0063	0.0066	0.0062
Stage 1–3 obs	6	6	6	6	6	6	6	6
Stage 1–3 null μ	0.999	0.991	0.988	0.984	1.012	1.002	1.007	0.987
Stage 1–3 null σ	0.884	0.877	0.881	0.886	0.887	0.880	0.880	0.867
Stage 1–3 z	5.654	5.709	5.692	5.648	5.624	5.677	5.674	5.778
Stage 1–3 p	< 0.001	< 0.001	< 0.001	< 0.001	< 0.001	< 0.001	< 0.001	< 0.001

Table S4: **Summary of statistical results for Aβ-based model seeding site predictions (ADNI).** Each column represents a subject group, and rows report observed seeding counts, null model means/standard deviations, z-scores, and p-values for Braak stage 1 and 1–3 regions.

Measure	CN	MCI	AD	All	Aβ^-	Aβ^+ τMTL$^-$ τNEO$^-$	Aβ^+ τMTL$^+$ τNEO$^-$	Aβ^+ τMTL$^+$ τNEO$^+$
Stage 1 obs	2	2	2	2	2	2	2	2
Stage 1 null μ	0.056	0.052	0.223	0.055	0.056	0.059	0.058	0.218
Stage 1 null σ	0.232	0.225	0.442	0.231	0.233	0.236	0.237	0.440
Stage 1 z	8.390	8.662	4.015	8.428	8.339	8.235	8.179	4.051
Stage 1 p	< 0.001	< 0.001	0.0112	< 0.001	< 0.001	< 0.001	0.0011	0.0115
Stage 1–3 obs	2	2	8	2	2	2	2	8
Stage 1–3 null μ	0.339	0.330	1.344	0.324	0.336	0.340	0.330	1.340
Stage 1–3 null σ	0.524	0.519	0.994	0.521	0.524	0.528	0.525	1.002
Stage 1–3 z	3.173	3.218	6.693	3.219	3.176	3.143	3.179	6.649
Stage 1–3 p	0.0251	0.0243	< 0.001	0.0259	0.0258	0.0274	0.0273	< 0.001

Regression statistics summary

We assessed whether model-derived tau seeding predictions correlate with empirical tau PET SUVRs using simple linear regression, with summarized statistics in Table S5. We also performed multiple linear regression with age, sex and APEO4 carrier status as covariates, both over all subjects and for each diagnosis group, with summarized statistics in Table S6.

Subgroup forest plots for multiple regression

To further explore diagnostic group differences in regression effects, we performed subgroup analyses for cognitively normal (CN), mild cognitive impairment (MCI), and Alzheimer's disease (AD) participants in the ADNI cohort. Figure S5 shows forest plots summarizing the standardized β estimates and 95% confidence intervals from the multiple regression models (including age, sex, and APOE4 as covariates) within each subgroup. Discrete explanatory variables were not standardized.

Model fit for multiple regression

To assess model assumptions and fit for the ADNI multiple regression analyses, we generated predicted-vs-observed and quantile-quantile plots for both FDG- and Aβ-based models. Figure S6 summarizes the results.

Table S5: **Simple linear regression summary (ADNI).** Simple regression results for model-predicted tau seeding versus empirical tau SUVR in entorhinal cortex (Stage 1) and Braak stages 2/3, across FDG-based and Aβ-based models in the ADNI cohort. N is the number of subjects; β is the slope estimate with 95% confidence interval (CI); r is the Pearson correlation coefficient.

Model	Stage	N	Pearson r	β [95% CI]	p-value	t
FDG-based	Stage 1	253	0.181	6.710 [2.168, 11.252]	0.0039	2.910
FDG-based	Stages 2/3	253	0.004	0.318 [-10.309, 10.945]	0.953	0.059
Aβ-based	Stage 1	453	0.398	11.257 [8.859, 13.656]	< 0.001	9.223
Aβ-based	Stages 2/3	453	0.397	18.956 [14.899, 23.012]	< 0.001	9.183

Table S6: **Multiple linear regression summary (ADNI).** Multiple regression results linking model-derived tau seeding predictions to empirical entorhinal tau PET SUVR in the ADNI cohort. Results are shown for FDG- and Aβ-based models across all subjects and diagnostic subgroups. β is the standardized slope of the model prediction term, with 95% confidence intervals (CI); r is the Pearson correlation coefficient for the full model. t_β and p_β refer specifically to the significance of the model prediction term.

Model	Group	Stage	N	r	F-test p	stand. β [95% CI]	t_β	p_β
FDG-based	All	1	253	0.535	< 0.001	0.191 [0.085, 0.298]	3.53	< 0.001
FDG-based	CN	1	14	0.694	0.16	-0.378 [-1.185, 0.429]	-1.06	0.32
FDG-based	MCI	1	170	0.541	< 0.001	0.208 [0.076, 0.339]	3.12	0.0021
FDG-based	AD	1	69	0.411	0.019	0.086 [-0.163, 0.335]	0.69	0.49
Aβ-based	All	1	453	0.446	< 0.001	0.342 [0.255, 0.429]	7.74	< 0.001
Aβ-based	CN	1	260	0.305	< 0.001	0.142 [0.019, 0.265]	2.27	0.024
Aβ-based	MCI	1	142	0.523	< 0.001	0.298 [0.149, 0.446]	3.96	< 0.001
Aβ-based	AD	1	51	0.457	0.027	0.140 [-0.130, 0.410]	1.04	0.30
FDG-based	All	2/3	253	0.428	< 0.001	0.060 [-0.058, 0.177]	1.00	0.31
FDG-based	CN	2/3	14	0.577	0.40	-0.505 [-1.388, 0.377]	-1.30	0.23
FDG-based	MCI	2/3	170	0.460	< 0.001	0.095 [-0.051, 0.241]	1.28	0.20
FDG-based	AD	2/3	69	0.428	0.012	0.282 [0.035, 0.528]	2.29	0.026
Aβ-based	All	2/3	453	0.426	< 0.001	0.362 [0.274, 0.451]	8.05	< 0.001
Aβ-based	CN	2/3	260	0.245	0.003	0.104 [-0.022, 0.229]	1.63	0.10
Aβ-based	MCI	2/3	142	0.498	< 0.001	0.296 [0.137, 0.455]	3.68	< 0.001
Aβ-based	AD	2/3	51	0.571	0.0010	0.243 [-0.017, 0.503]	1.88	0.067

Regression results for Braak Stage 2/3

We also performed regression analyses using empirical averaged tau-PET SUVR in Braak Stage 2/3 regions as the outcome. Figure S7 summarizes the results for the ADNI cohort.

Replication cohort: Harvard Aging Brain Study

To assess the robustness of our computational seeding model, we replicated our analyses using the HABS cohort. This replication encompassed FDG-based and Aβ-based seeding predictions, null tests, and subject-level correlation analyses, as summarized in Fig. S8. The results are similar to those observed in the ADNI cohort, with the computational model predicting tau seeding primarily in the entorhinal cortex for FDG- and Aβ-based predictions, as seen in Fig. S8A,B. Permutation tests confirmed significant entorhinal seeding for both FDG- and Aβ-based models (p = 0.030 and p = 0.0018, respectively, Fig. S8C). We also observed Aβ-based seeding predictions in the pars orbitalis in the HABS cohort, which were not present in the ADNI results. Full statistics on the permutation tests are provided in Table S7. Aβ-based predictions exhibited modest subject-level correlations with empirical entorhinal tau (r = 0.29, unstandardized β = 3.25, 95% CI 1.92–4.58, p < 0.001, n = 255; Fig S8D, see Table S8 for detailed statistics), while no correlation were found for FDG-based predictions (r = -0.024, unstandardized β = -0.43, 95% CI -3.81–2.94, p = 0.80, n = 116). Adjusting for age, sex, and APOE4 status in a multiple linear model similarly showed significant correlation between Aβ-based model predictions and empirical entorhinal tau (standardized β = 0.17, p = 0.0088), but not for the FDG-based model (see Fig. S11–S12; full statistics in Table S9). Aβ-based predictions are also correlated to Braak stages

Table S7: **Summary of statistical results for FDG- and Aβ-based model seeding site predictions (HABS).** Each column reports the observed number of seeded regions, null distribution mean and standard deviation, z-score, and p-value for Braak stage 1 (entorhinal cortex) and stages 1–3.

Measure	FDG All	Aβ all
Stage 1 obs	1	2
Stage 1 null μ	0.030	0.109
Stage 1 null σ	0.170	0.317
Stage 1 z	5.696	5.963
Stage 1 p	0.0299	0.0018
Stage 1–3 obs	1	2
Stage 1–3 null μ	0.169	0.656
Stage 1–3 null σ	0.375	0.724
Stage 1–3 z	2.218	1.858
Stage 1–3 p	0.169	0.124

2/3 empirical tau both in simple (r = 0.39, $p < 0.001$) and multiple linear regression (standardized $\beta = 0.26$, $p < 0.001$; see Fig. S13). To assess whether the smaller HABS sample size (n = 116) compared to ADNI (n = 253) could explain the lack of significance in the FDG-based predictions, we performed a statistical power test; we subsample 116 samples from the ADNI FDG-tau scan pairs and repeat the simple linear regression analysis (100,000 iterations) to estimate how likely we are to get significant correlations $p < 0.05$ with the HABS cohort size. As shown in Fig. S14, the HABS cohort size only recovers significance 49% of the time, indicating low statistical power. In addition, entorhinal tau SUVR values show smaller variability in HABS compared to ADNI, suggesting that the narrower distribution in HABS may have further limited our ability to detect subject-level FDG-based associations, as seen in Fig. S15.

PET data processing (HABS)

We replicated our analyses in the Harvard Aging Brain Study cohort.36 HABS data version 3.0 was obtained in April 2025 via synapse.org. Publicly available summary tables were used, which include PET-derived SUVRs mapped to Desikan–Killiany atlas regions, computed from images processed with FreeSurfer v6.0 and PETSurfer. All scans had undergone quality control and were registered to the subject's T1-weighted MRI using a 6-DOF rigid-body transformation in SPM12. Because HABS participants were cognitively normal at enrollment, we selected the latest available PET scan per participant. Given that the HABS cohort is largely cognitively normal, stratified analyses by clinical diagnosis or biomarker status were not pursued. Partial-volume correction was not applied. Aβ and tau PET scans were normalized using the bilateral cerebellar gray matter as the reference region, while the FDG PET were normalized to the pons. Similarly to the ADNI PET data, spurious signal in regions with known susceptibility to segmentation and registration artifacts were replaced with the mean itself and their anatomical neighbors (see Table S1).[35] The averaged PET data are shown in Fig. S9.

Null model for seeding susceptibility (HABS)

The null distributions for the aggregated seeding predictions under randomly shuffled PET data for the HABS cohort are shown in Fig. S10. The corresponding statistics for the HABS dataset is found in Table S7.

Regression statistics (HABS)

In the HABS cohort, we also assessed whether subject-level predictions aligned with empirical tau in early Braak regions using simple linear regression, with statistics summarized in Table S8.

Model fit for multiple regression (HABS)

We also evaluated model fit for the HABS multiple regression analyses using Q–Q plots and predicted-versus-observed comparisons. Figure S11 presents the diagnostic results for both FDG- and Aβ-based models.

Table S8: **Simple linear regression summary (HABS)**. Simple regression results for model-predicted tau seeding versus empirical tau SUVR in entorhinal cortex (Stage 1) and Braak stages 2/3, across FDG-based and Aβ-based models in the HABS cohort. N is the number of subjects; β is the raw (not standardized) slope estimate with 95% confidence interval (CI); r is the Pearson correlation coefficient; t is the t-statistic for β_1.

Model	Stage	N	Pearson r	β [95% CI]	p-value	t
FDG-based	Stage 1	116	-0.024	-0.433 [-3.806, 2.940]	0.799	-0.254
FDG-based	Stages 2/3	116	-0.000	-0.008 [-5.064, 5.049]	0.997	-0.003
Aβ-based	Stage 1	255	0.290	3.247 [1.918, 4.576]	< 0.001	4.812
Aβ-based	Stages 2/3	255	0.386	7.011 [4.937, 9.084]	< 0.001	6.658

Table S9: **Multiple linear regression summary (HABS).** Multiple regression results linking model-derived tau seeding predictions to empirical entorhinal tau PET SUVR in the HABS cohort. Results are shown for FDG- and Aβ-based models across all subjects and diagnostic subgroups. β is the standardized slope of the model prediction term, with 95% confidence intervals (CI); r is the Pearson correlation coefficient for the full model. t_β and p_β refer specifically to the significance of the model prediction term.

Model	Group	Stage	N	r	F-test p	stand. β [95% CI]	t_β	p_β
FDG-based	All	1	116	0.481	< 0.001	-0.007 [-0.173, 0.160]	-0.08	0.94
FDG-based	CN	1	115	0.463	< 0.001	-0.025 [-0.195, 0.144]	-0.29	0.77
FDG-based	MCI	1	1	-	-	- [-, -]	-	-
FDG-based	AD	0	-	-	-	- [-, -]	-	-
Aβ-based	All	1	255	0.382	< 0.001	0.172 [0.044, 0.301]	2.64	0.0088
Aβ-based	CN	1	238	0.303	< 0.001	0.080 [-0.059, 0.219]	1.14	0.26
Aβ-based	MCI	1	12	0.594	0.49	0.132 [-0.674, 0.938]	0.39	0.71
Aβ-based	AD	1	5	-	-	- [-, -]	-	-
FDG-based	All	2/3	116	0.457	< 0.001	0.005 [-0.162, 0.172]	0.06	0.95
FDG-based	CN	2/3	115	0.436	< 0.001	-0.003 [-0.173, 0.167]	-0.04	0.97
FDG-based	MCI	2/3	170	0.459	< 0.001	0.093 [-0.053, 0.239]	1.26	0.21
FDG-based	AD	2/3	69	0.428	0.011	0.281 [0.035, 0.527]	2.28	0.026
Aβ-based	All	2/3	255	0.464	< 0.001	0.264 [0.136, 0.393]	4.06	< 0.001
Aβ-based	CN	2/3	238	0.394	< 0.001	0.180 [0.044, 0.317]	2.61	0.0097
Aβ-based	MCI	2/3	12	0.622	0.42	0.600 [-0.617, 1.818]	1.17	0.28
Aβ-based	AD	2/3	5	-	-	- [-, -]	-	-

Multiple regression results (HABS)

To evaluate the relationship between model-derived seeding predictions and entorhinal tau in the HABS cohort, we conducted multiple linear regression analyses including age, sex, and APOE4 status as covariates. Figure S12 summarizes the results for both FDG- and Aβ-based models.

Regression results for Braak Stage 2/3 (HABS)

To complement the ADNI results, we conducted regression analyses using empirical tau-PET SUVR in Braak Stage 2/3 regions in the HABS cohort. Figure S13 summarizes the results.

Statistical power analysis for HABS cohort

To determine whether the lack of a significant FDG-based correlation in the HABS cohort could be attributed to limited sample size, we performed a subsampling-based power analysis of the ADNI samples (see Fig. S14). Specifically, we repeatedly drew $n = 116$ random subsamples (matching the HABS FDG/tau sample size) from the ADNI FDG–tau scan pairs and computed the subject-level correlation between predicted and empirical tau SUVR in the entorhinal cortex. This procedure was repeated 100,000 times to estimate how frequently a significant correlation ($p < 0.05$) could be recovered given HABS-level statistical power.

Distribution of entorhinal tau SUVR in ADNI and HABS

To explore whether differences in tau-PET signal variability between cohorts may help explain the differing FDG-based regression results, we visualized the distributions of empirical entorhinal tau SUVR values in both ADNI and HABS. As shown in Figure S15, the ADNI cohort exhibits a broader and more right-skewed distribution, reflecting greater inter-subject variability and a higher proportion of individuals with elevated tau. By contrast, the HABS cohort shows a more compact distribution, with most individuals clustering around lower SUVR values. These differences in signal variability may partially account for the lack of a detectable FDG-based correlation in HABS.

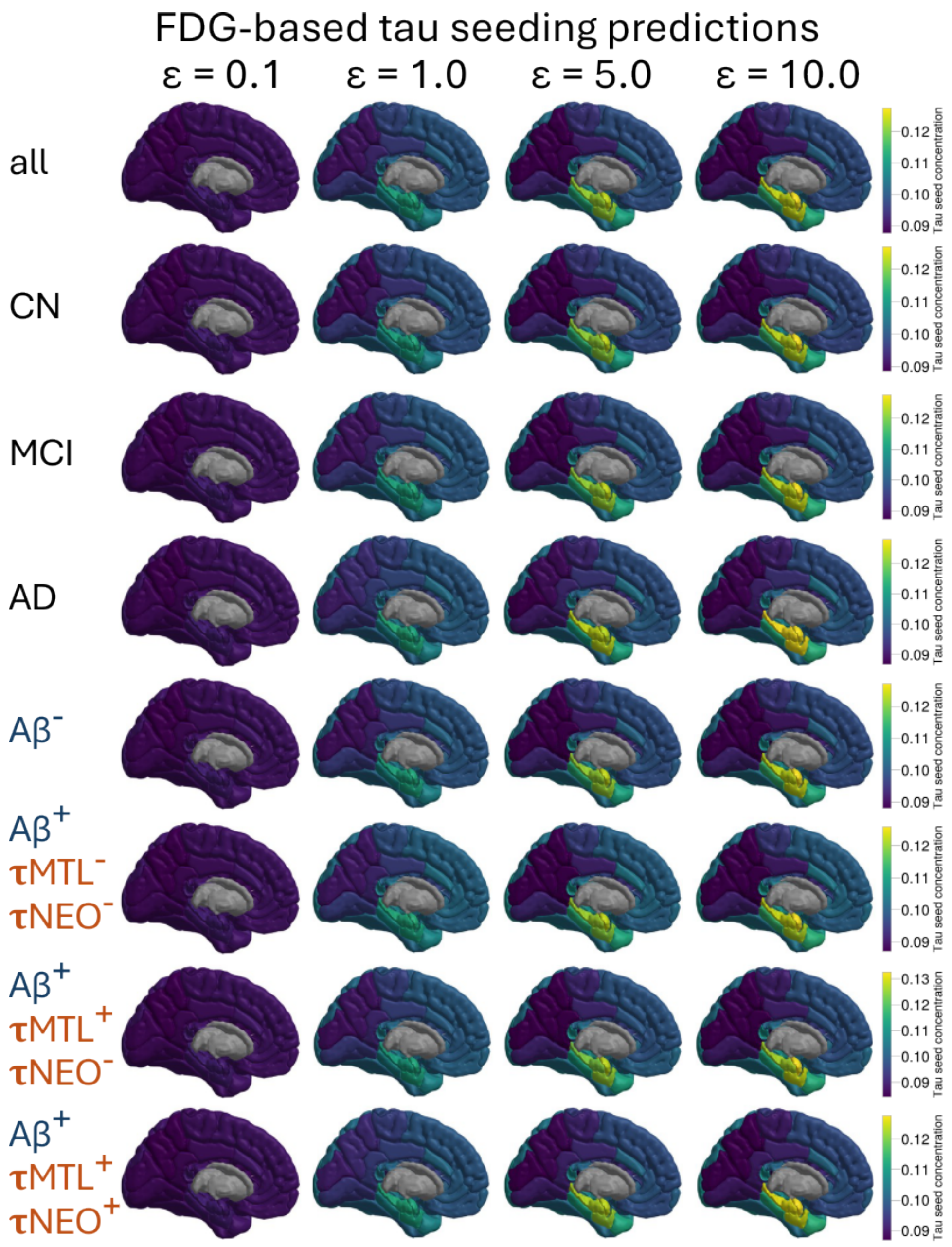

Figure S2: **Predicted tau seeding concentrations across subject groups and FDG impact levels (ADNI).** Rows correspond to subject groups stratified by diagnosis and biomarker status. Columns represent increasing levels of the impact of metabolic activity (FDG) on tau seeding, progressing from left to right.

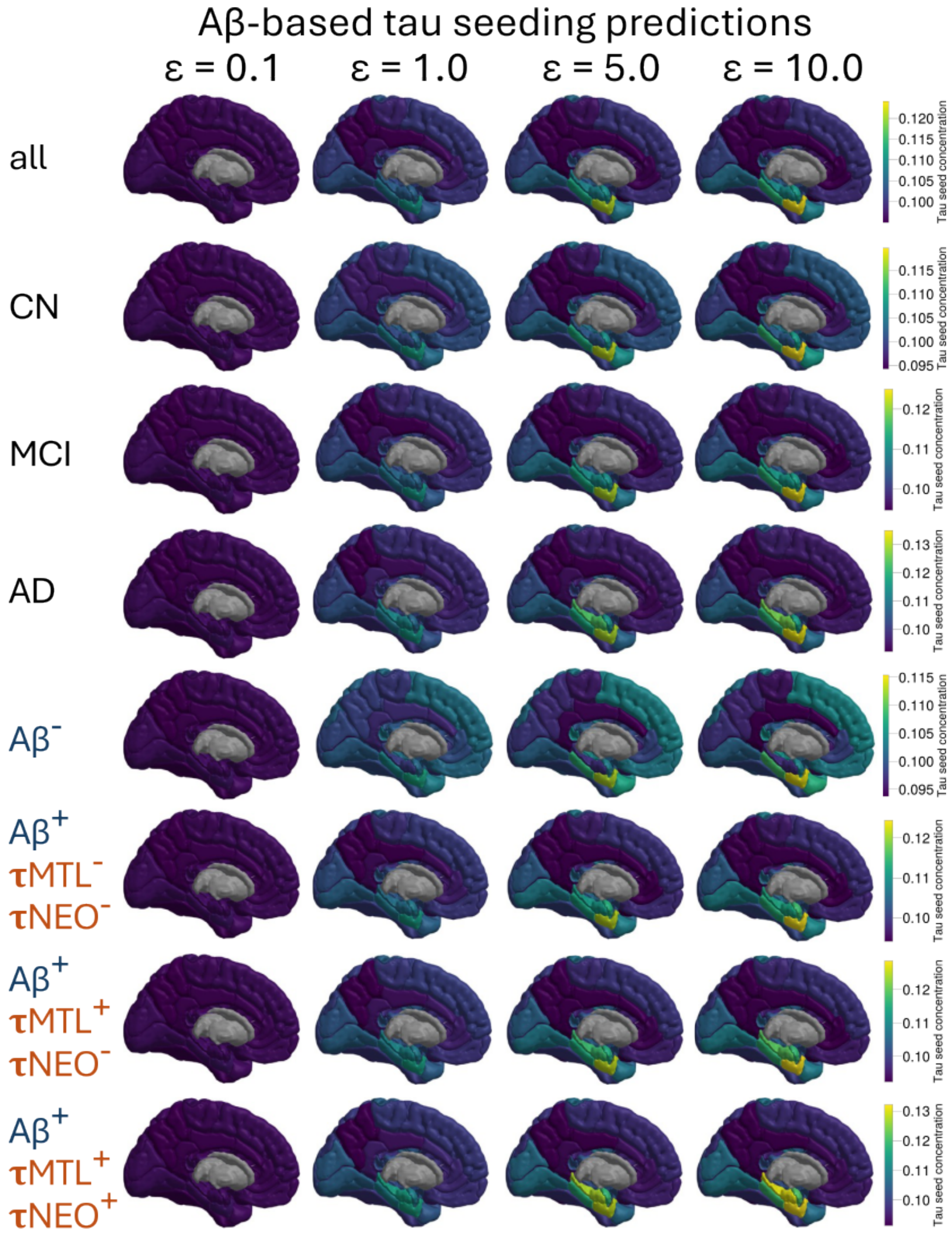

Figure S3: **Predicted tau seeding concentrations across subject groups and Aβ impact levels (ADNI).** Rows correspond to subject groups stratified by diagnosis and biomarker status. Columns represent increasing levels of the predicted impact of Aβ-deposition on tau seeding, progressing from left to right.

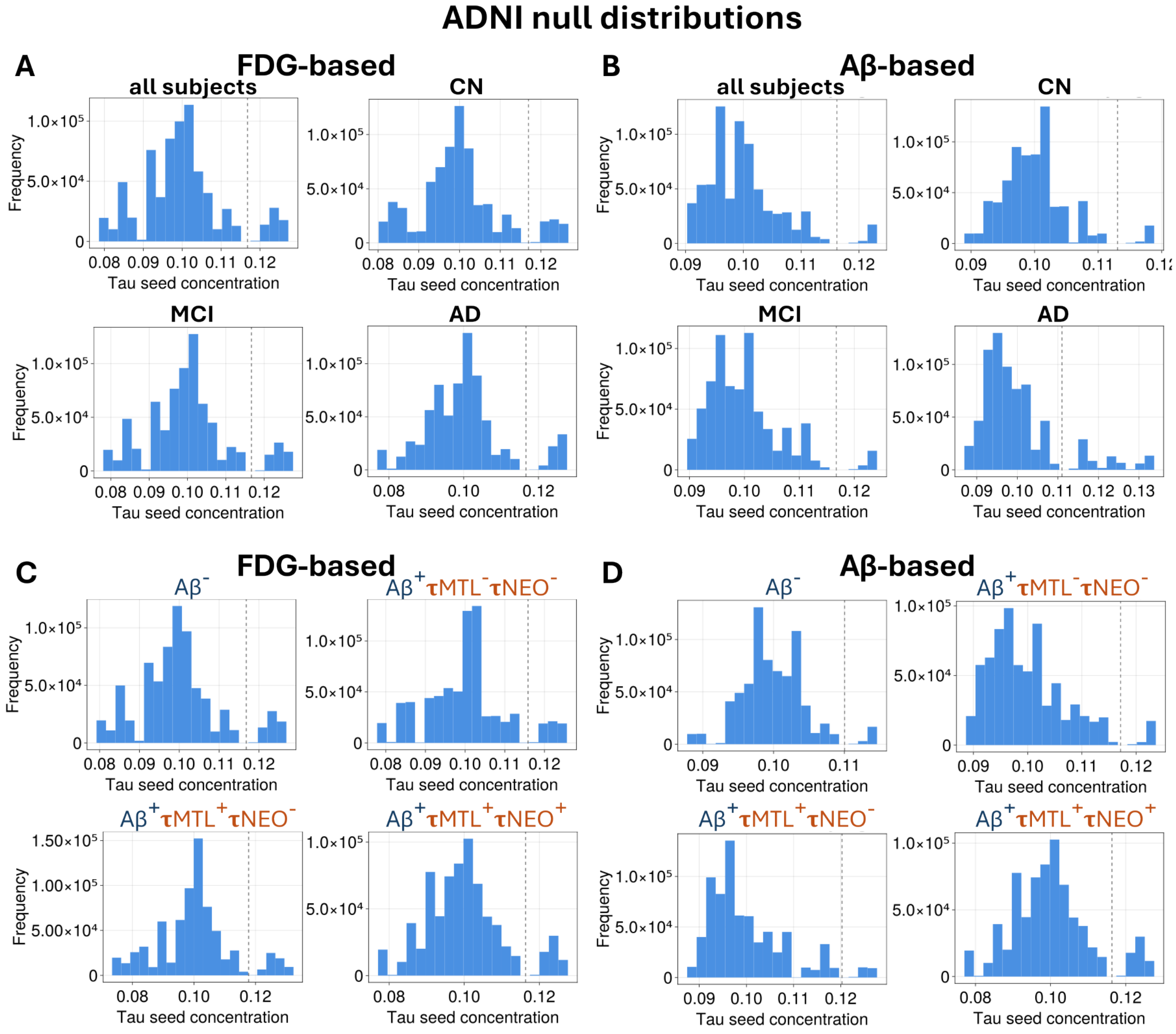

Figure S4: **Null model distributions for seeding region predictions (ADNI).** Histograms of seeding concentrations pooled across all brain regions and trials (10,000) in the null model. **(A,B)** Null distributions for FDG- and Aβ-based models stratified by clinical diagnosis. **(C,D)** Null distributions for the same models stratified by biomarker-defined groups. In each case, seeding concentrations are derived from shuffled PET maps, and the seeding threshold (grey stippled line) is defined by the midpoint of the largest gap in the distribution above the median.

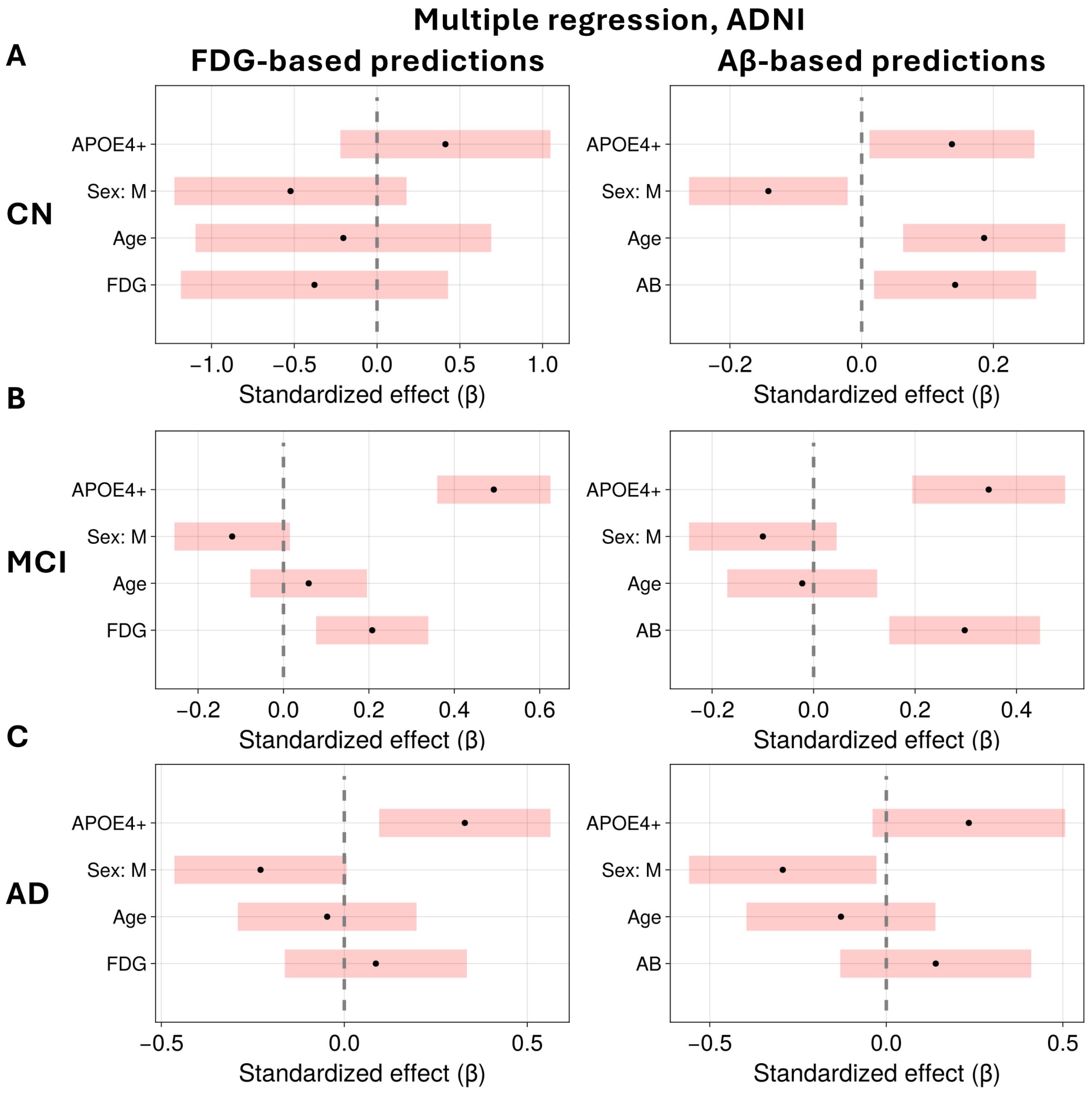

Figure S5: **Subgroup forest plots from multiple regression analyses (ADNI). (A)** Cognitively normal (CN), **(B)** mild cognitive impairment (MCI), and **(C)** Alzheimer's disease (AD) groups. Each panel shows standardized β estimates and 95% confidence intervals for FDG-based (left) and Aβ-based (right) predictions, adjusted for age, sex, and APOE4 status.

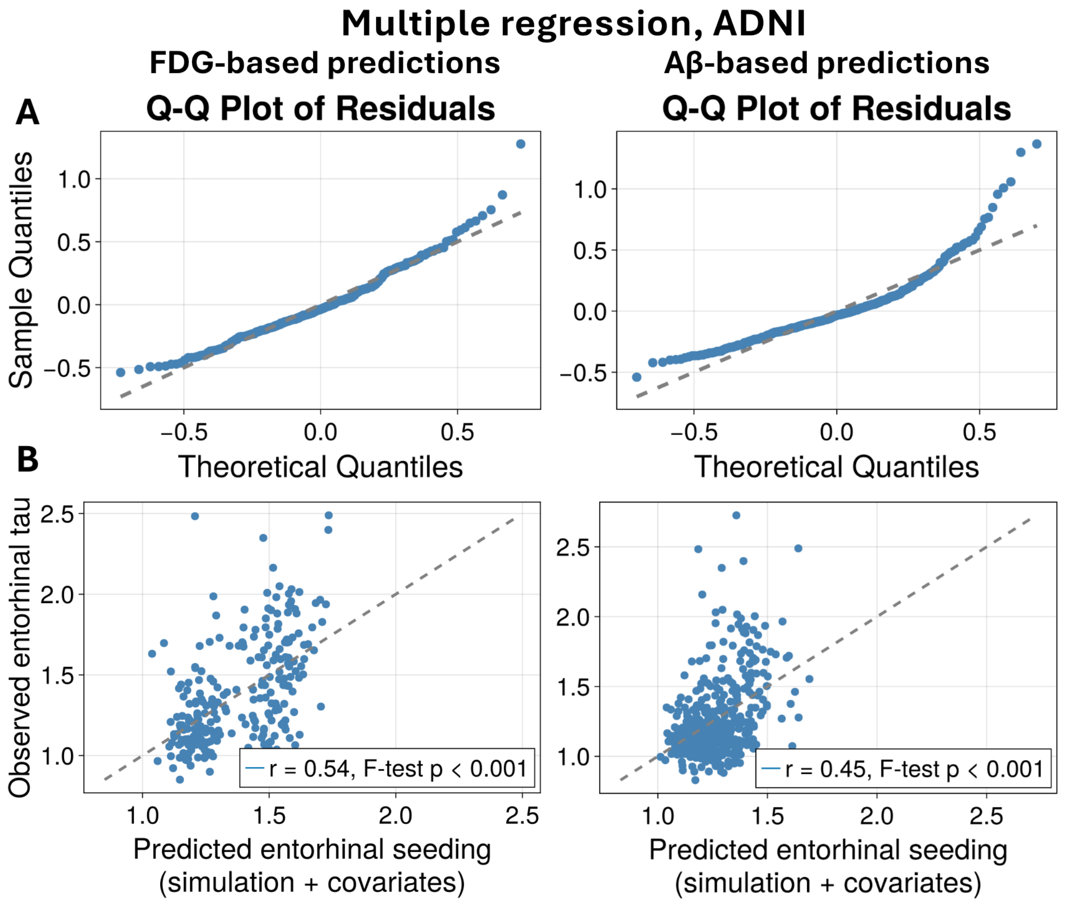

Figure S6: **Model diagnostics for multiple regression (ADNI). (A)** Q–Q plots comparing predicted and observed entorhinal tau SUVR values for FDG-based (left) and Aβ-based (right) models. **(B)** Predicted versus observed tau SUVR values for the same models based on the full multiple regression, including age, sex, and APOE4 as covariates.

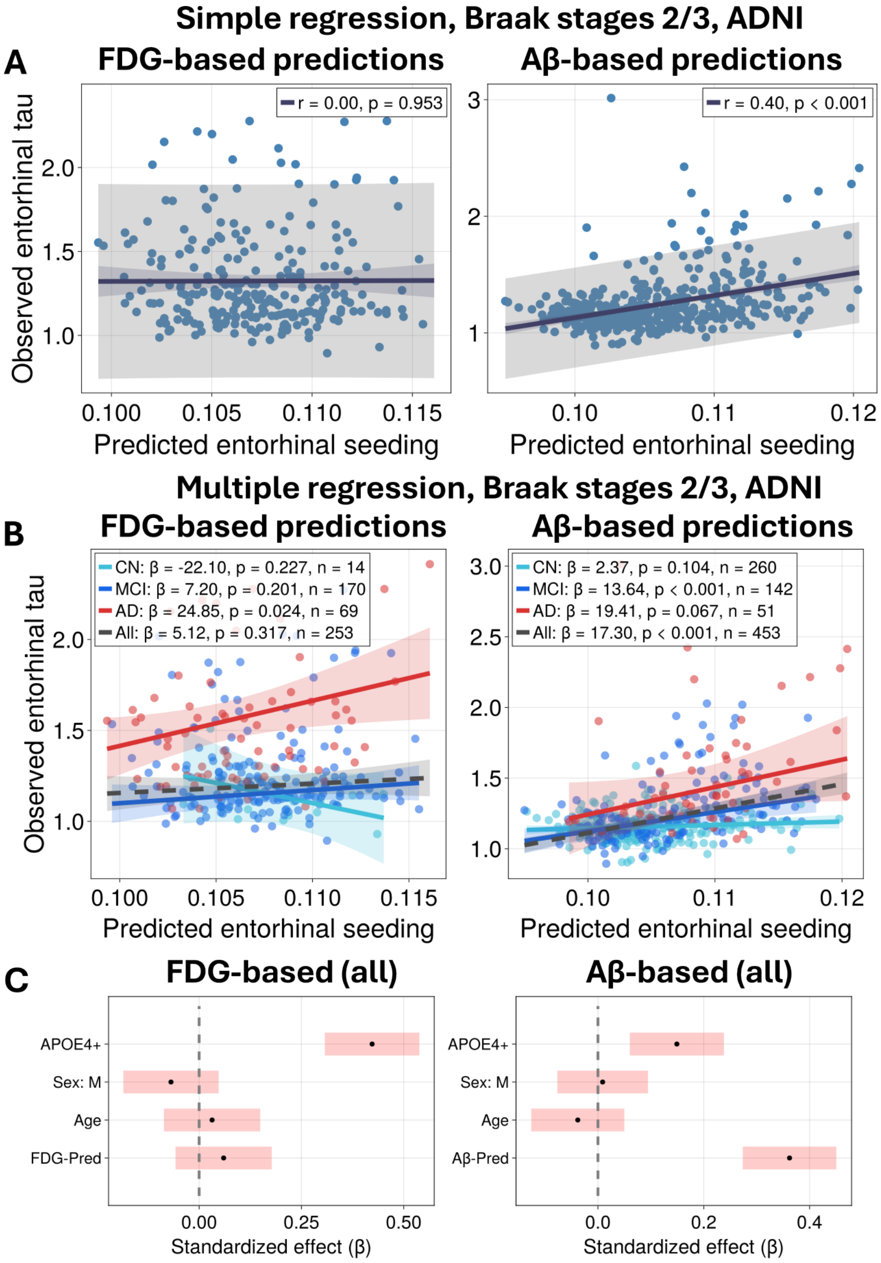

Figure S7: **Regression results for Braak Stage 2/3 (ADNI). (A)** Simple linear regressions between model-derived seeding values and empirical tau SUVR for FDG-based (left) and Aβ-based (right) models. **(B)** Marginalized effect plots from multiple linear regression including age, sex, and APOE4 covariates. Group-level estimates are shown. Legend shows unstandardized β estimates, p-values for the model prediction term, and number of samples. **(C)** Forest plot showing standardized β estimates and 95% confidence intervals for the full sample (all subjects) in the multiple regression model.

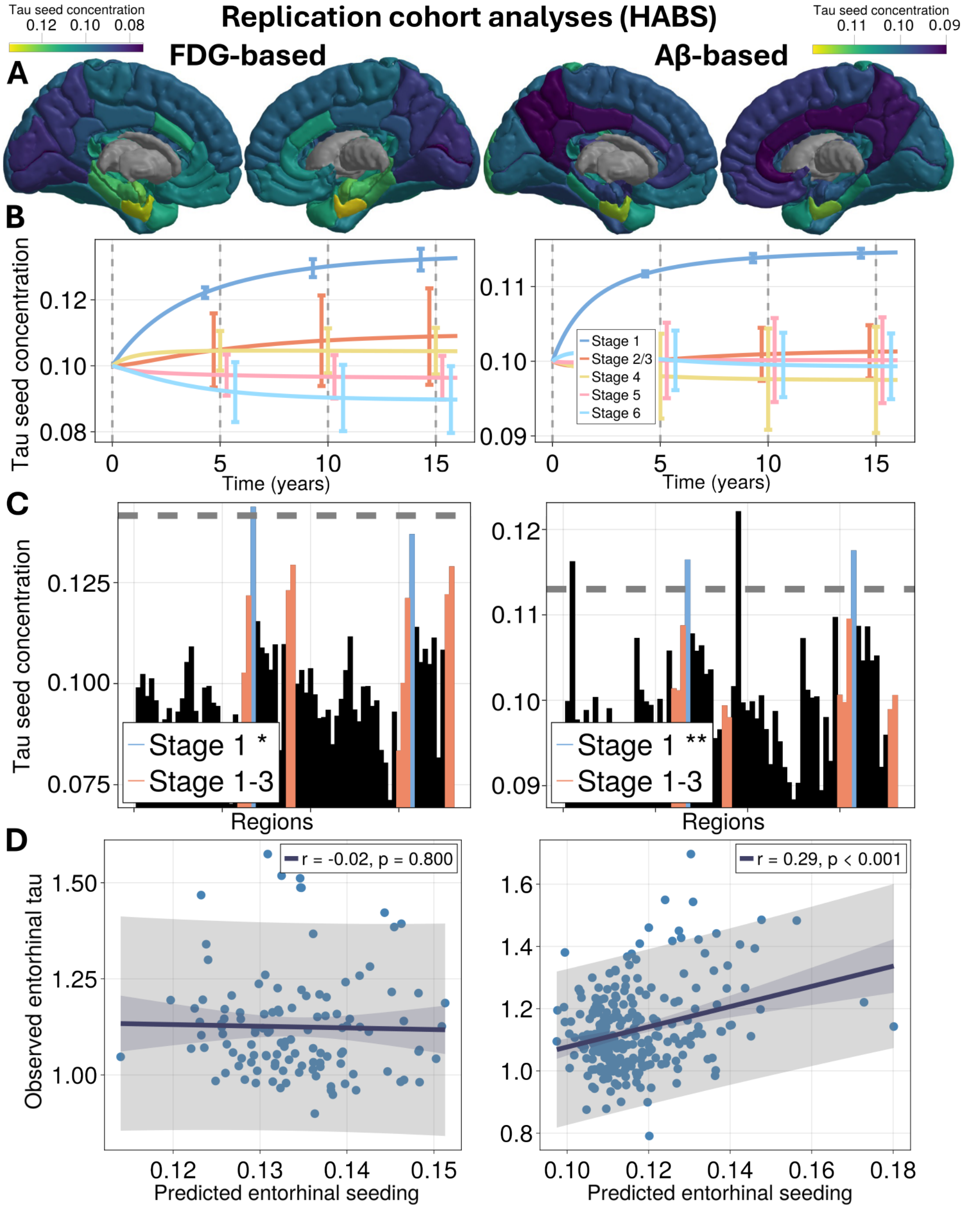

Figure S8: **Summary of replicated analysis using the Harvard Aging Brain Study cohort.** This figure presents results from FDG-based (left-column, all rows) and Aβ-based (right-column, all rows) seeding predictions, null tests, and subject-level correlation analyses. (A) Predicted steady-state tau seed concentrations mapped onto a 3D brain surface. (B) Temporal evolution of tau seed concentrations averaged over Braak stages. (C) Statistical testing of predicted tau seeding regions using a permutation null model. (D) Subject-level correlation analyses (simple regression) between model-derived seeding predictions and empirical tau levels in the entorhinal cortex. Modeling parameters are as described in Materials and methods.

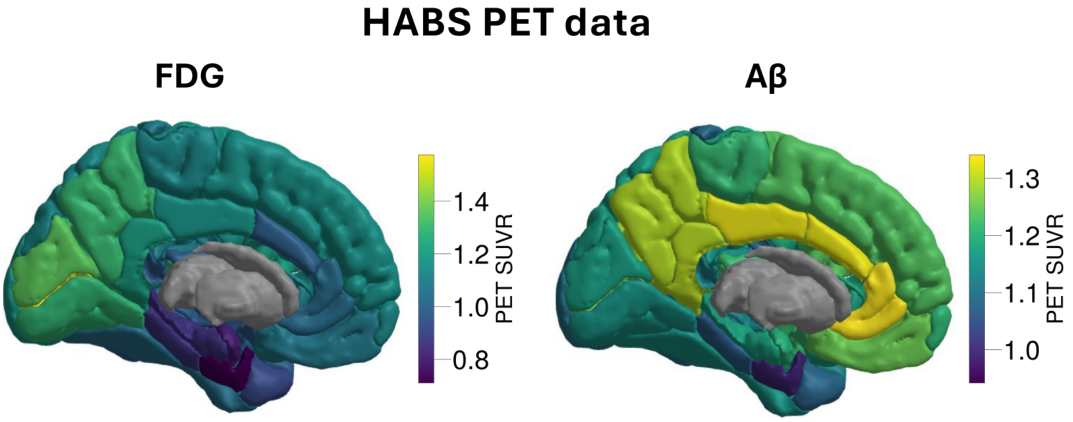

Figure S9: **Group-averaged PET SUVRs (HABS).** Left: FDG-PET SUVR map averaged across all HABS subjects. Right: amyloid-β PET SUVR map averaged across the same cohort. Each map displays the average left-hemisphere cortical SUVRs projected onto a common surface.

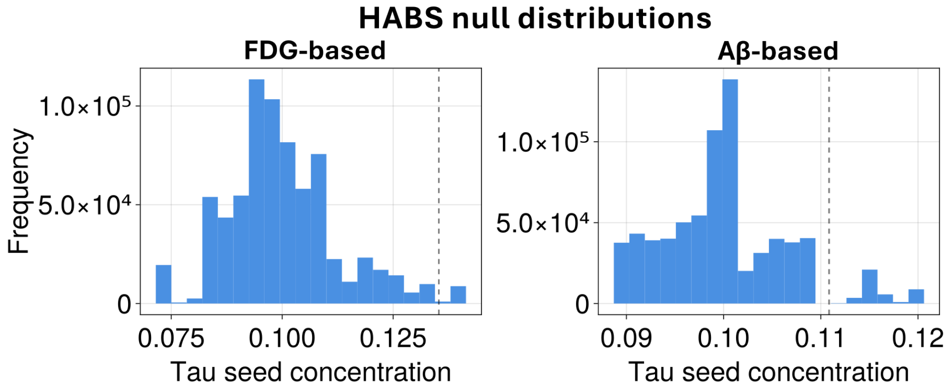

Figure S10: **Null model distributions for seeding region predictions (HABS).** Histograms show the distribution of seeding concentrations across all regions and 10,000 null model trials. Left and right panels correspond to FDG- and Aβ-based models, respectively. Seeding concentrations are derived from shuffled PET maps, and the seeding threshold (grey stippled line) is defined as the midpoint of the largest gap in the distribution above the median.

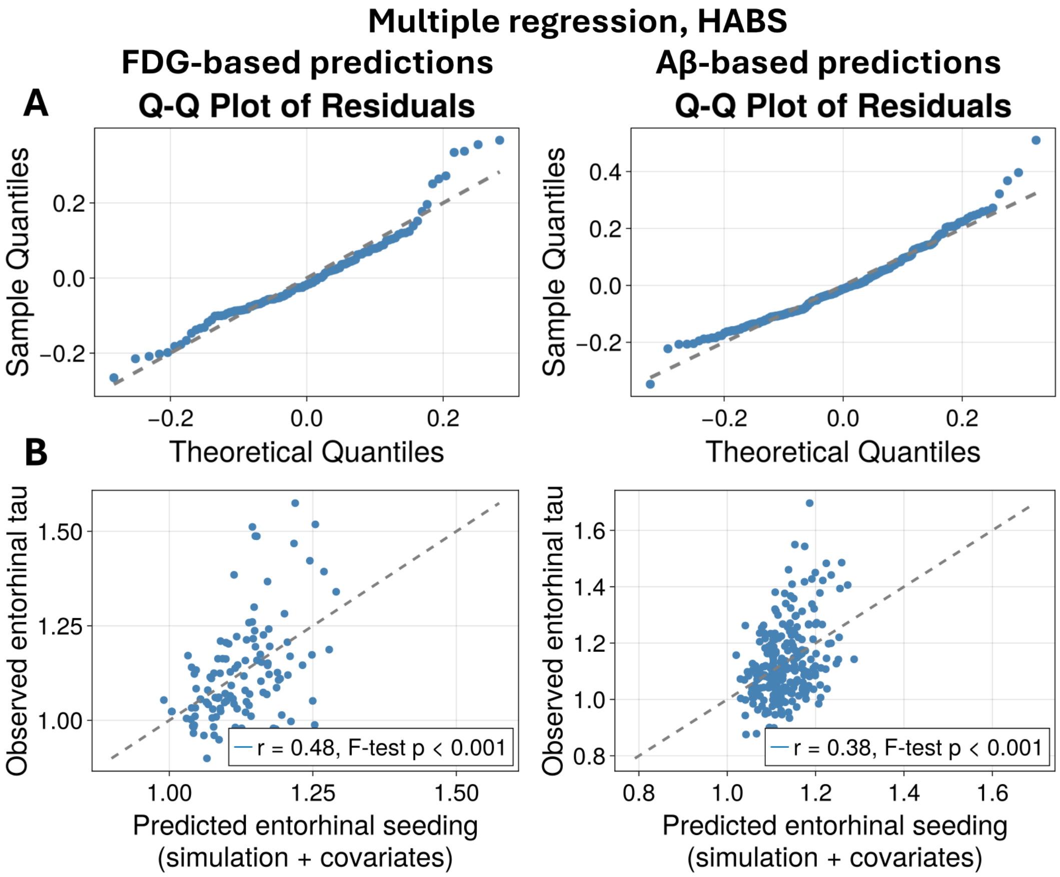

Figure S11: **Model diagnostics for multiple regression (HABS). (A)** Q–Q plots comparing predicted and observed entorhinal tau SUVR values for FDG-based (left) and Aβ-based (right) models. w**(B)** Predicted versus observed tau SUVR values from the multiple regression models including age, sex, and APOE4 as covariates.

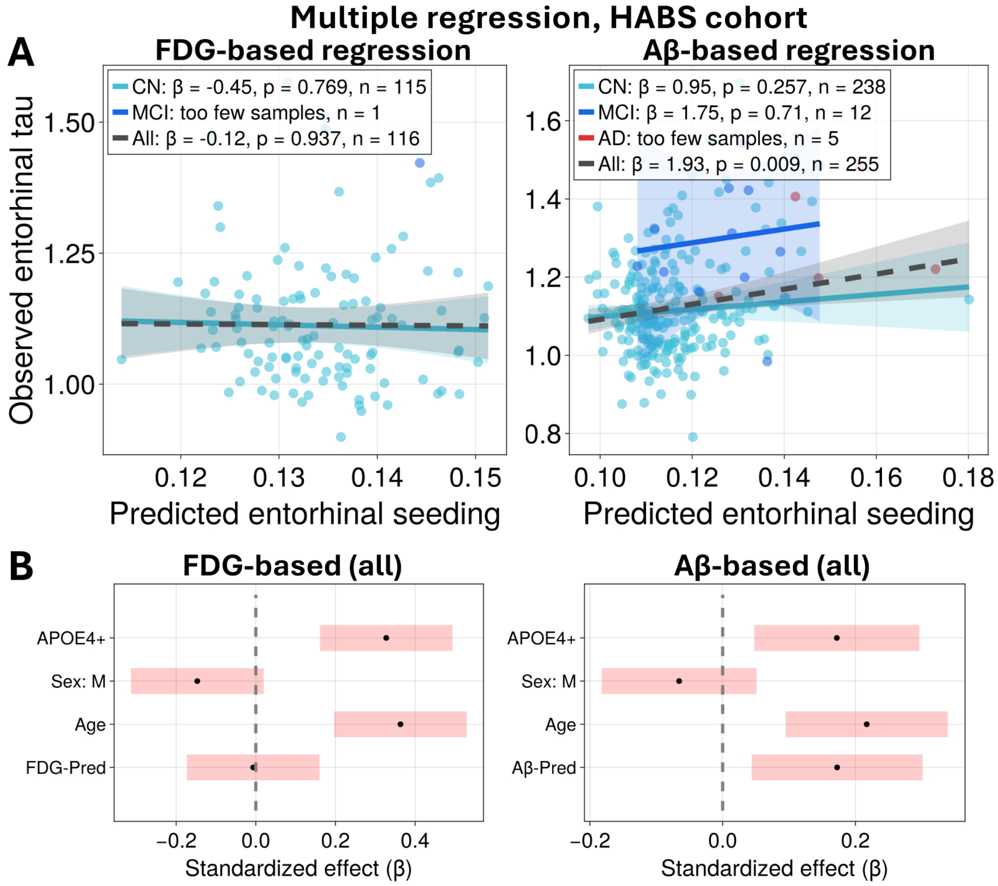

Figure S12: **Multiple regression (HABS). (A)** Marginalized effect plots from multiple linear regression including age, sex, and APOE4 covariates. Shown are estimates across diagnostic subgroups for FDG-based (left) and Aβ-based (right) predictors; the AD group is omitted from the FDG-based model due to lack of AD participants. Legend shows unstandardized β estimates, p-values for the model prediction term, and number of samples. **(B)** Forest plots for the full HABS sample showing standardized β estimates (black points) and their 95% confidence intervals (red-shaded region).

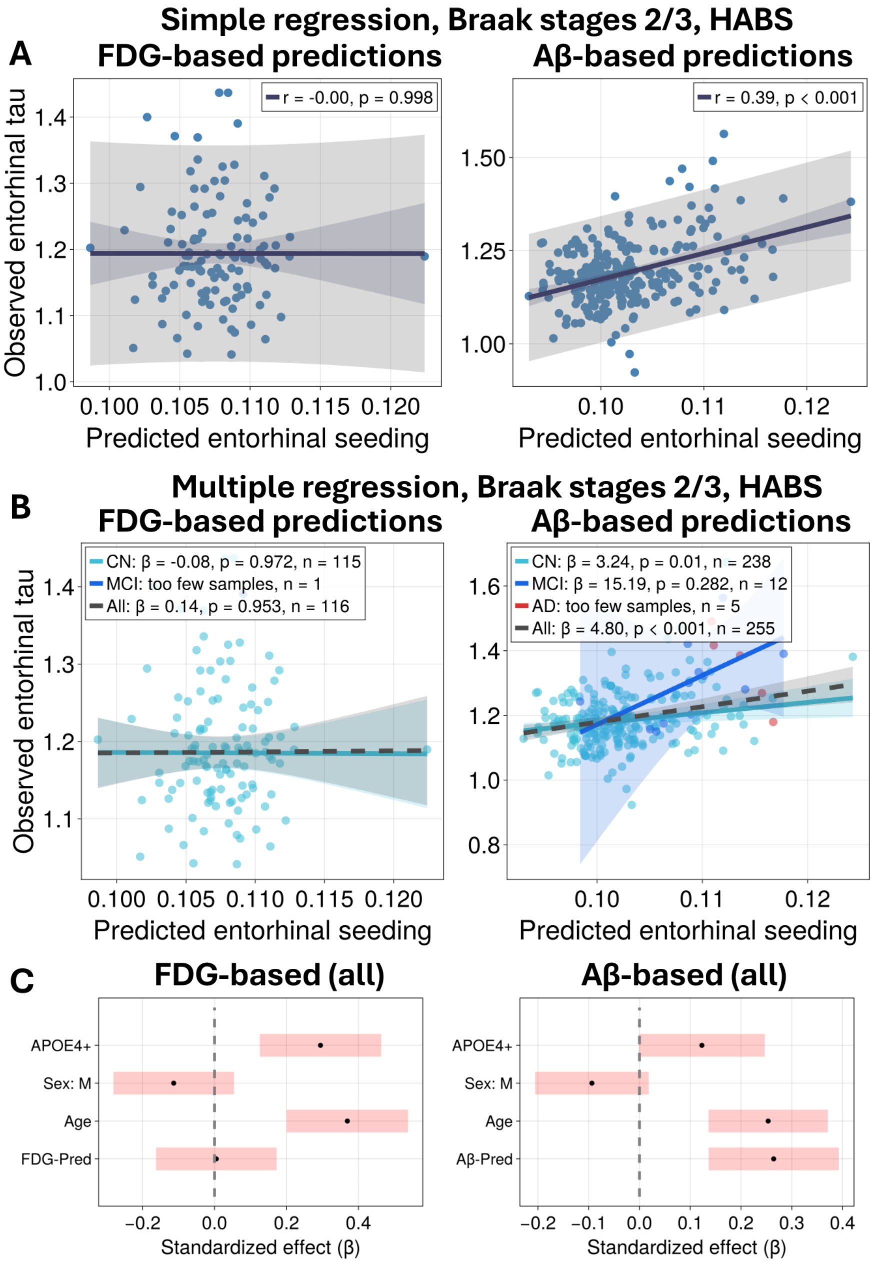

Figure S13: **Regression results for Braak Stage 2/3 (HABS). (A)** Simple linear regressions between model-derived seeding values and empirical tau SUVR for FDG-based (left) and Aβ-based (right) models.**(B)** Marginalized effect plots from multiple linear regression including age, sex, and APOE4 covariates. Group-level estimates are shown; groups with insufficient data to estimate effects are marked as "too few." The AD group is omitted from the FDG-based plot due to a lack of AD participants in the HABS FDG–tau dataset. Legend shows unstandardized β estimates, p-values for the model prediction term, and number of samples. **(C)** Forest plots for the full HABS sample showing standardized β estimates (black points) and their 95% confidence intervals (red-shaded region).

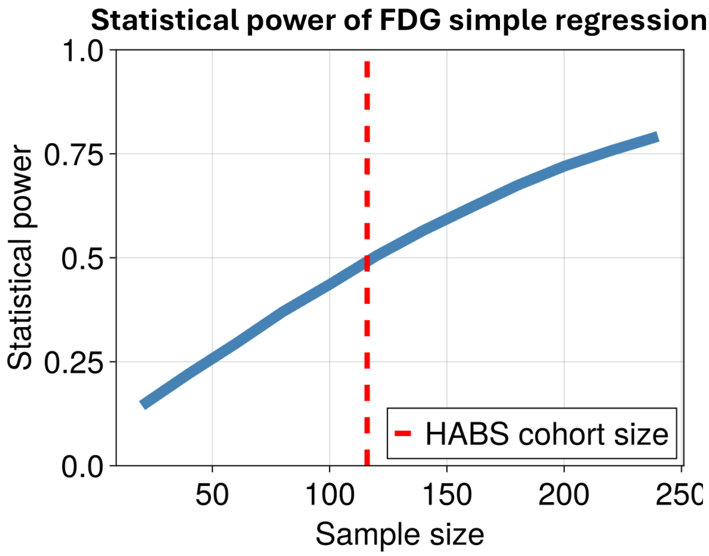

Figure S14: **Statistical power analysis for FDG-based simple regression in HABS.** Histogram shows the distribution of p-values across 100,000 subsampled correlations using $n = 116$ draws from ADNI. The proportion of significant correlations ($p < 0.05$) was 49%, indicating low power to replicate the ADNI FDG-based result in a sample the size of HABS. The proportion for the original ADNI sample size (n=251) was 81%.

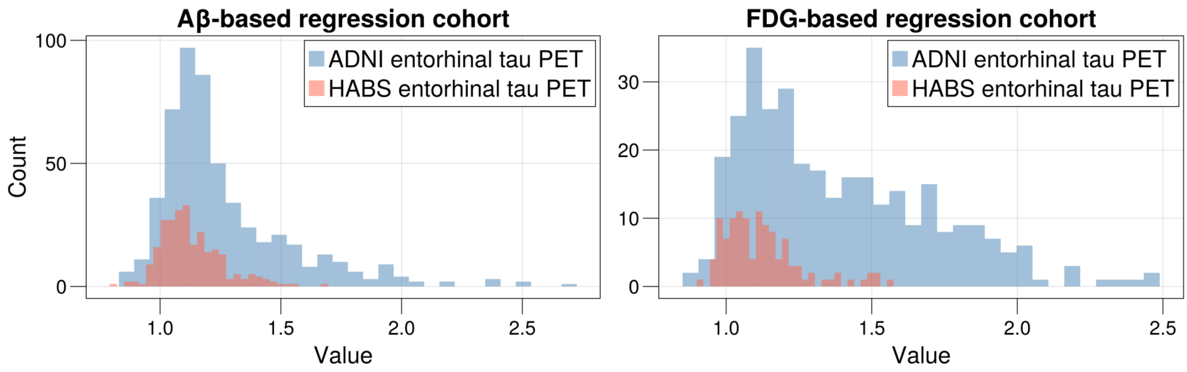

Figure S15: **Distributions of empirical entorhinal tau SUVR values in ADNI and HABS subcohorts used in regression analyses.**
